# TCF7 is highly expressed in immune cells on the atherosclerotic plaques, and regulating inflammatory signaling via NFκB/AKT/STAT1 signaling

**DOI:** 10.1042/BSR20212064

**Published:** 2022-07-18

**Authors:** Zhongnan Ma, Chuang Wang, Xiufeng Bai, Long Wang, Qianjing Wu, Zehong Cai, Wanxiang Wang, Zhuo Ma, Xinyu Liu, Jiaxuan Feng, Rui Feng

**Affiliations:** 1 Department of Vascular Surgery, Intervention Center, Shanghai General Hospital, Shanghai Jiao Tong University, Shanghai 200080, China; 2 Department of Laboratory of Human Diseases and Immunotherapies, State Key Laboratory of Biotherapy, West China Hospital, Sichuan University, Chengdu 610041, China; 3 Department of Research & Development, GenEros BioPharma Ltd., Hangzhou 310018, China; 4 Department of Biology, China Pharmaceutical University, Nanjing 210009, China

**Keywords:** Atherosclerosis, inflammation, TCF7, XCL1

## Abstract

Atherosclerosis, which is the fundamental basis for cardiovascular diseases in the global world, is driven by multiple roles of the immune system in the circulation and vascular plaque. Recent studies demonstrated that T-cell infiltrates into aorta plaque and plays an important role in recruiting macrophages to the vascular wall. Here, using single-cell sequencing, we found T cells in patients’ plaques and differentially expressed genes (DEGs) of T cells in atherosclerosis mice. T cells and macrophages were continuously activated in atherosclerotic plaque in patients. Besides, other immune cells also take part in atherogenesis, such as natural killer (NK) cells, granulocytes. Interferon (IFN)/NFκB signaling, the AKT signaling pathway was highly activated in mouse (*in vivo*) and cell line (*in vitro*). TCF7 and XCL1 were regulated by AKT and NFκB, respectively through protein–protein network analysis. Therefore, we attempt to clarify and discover potential genes and new mechanisms associated with atherosclerosis for drug development.

## Introduction

Atherosclerosis and its complications are the leading cause of death worldwide (myocardial infarction, stroke) [1,2]. Previous studies have shown that atherosclerosis is an inflammatory disease. And activated immune cells are the key components of atherosclerotic plaques, leading to plaque instability and clinical cardiovascular events [3–5].

There is little known about immune mechanisms underlying atherosclerosis. Histological studies of patients’ plaque and mouse models have shown that atherosclerotic plaque consisted of large necrotic cores, thin fibrous caps, and high proportions of macrophages [6]. Thus, macrophages were characterized as drivers of plaque instability by most researchers [5,7]. However, the diversity of immune cells in atherosclerotic plaques suggests different functions of other immune cells in plaque. For instance, T cells (T helper 1 or Treg cells) located in atherosclerotic plaque could aggravate or attenuate atherosclerosis in mouse models [8], but the contribution of T cells in the pathogenesis of atherogenesis remained unclear. JAK-STAT signaling pathway is vital important for immune cell development and inflammation [9,10]. Several studies have shown that circulating immune cells have a positive effect on the progression of atherosclerosis, such as the increase in monocytes and CD4^+^ T-cell subtypes [11–13]. However, the cross-talk of the immune response is still being studied. The phenotype and functional relationship of immune cells in atherosclerotic plaques and PBMC (peripheral blood mononuclear cell) are of great significance for disease prevention and treatment. Therefore, we want to identify potential therapeutic targets by differentially expressed genes (DEGs) in sequencing data.

Nonetheless, the approaches used to define the identities of T cells and macrophages need to cover the shortage because they came from a single source and used a limited range of specific stimuli *in* *vitro*. It did not reflect the complexity of the plaque microenvironment [14–16]. Thus, the phenotypic characteristics and heterogeneity of leukocyte infiltration in atherosclerotic plaques still need to be determined by systematic studies of cells directly derived from the pathological vascular microenvironment.

Drugs targeting inflammation or cholesterol synthesis are used to alleviate the symptom of atherosclerosis, such as *Atorvastatin* (a cholesterol synthesis inhibitor) and *Canakinumab* (an anti-inflammatory compound) [17,18], *Alirocumab* (PCSK9 inhibitor) [19], but only as part of patients’ response to the treatment, suggesting the necessity of novel target to discover the multifaceted nature of atherosclerotic formation. Knockout of inflammatory genes reduced atherosclerosis and alleviated hypercholesterolemia in mice treatment of targeting immune responses such as interleukin-1β (IL-1β) inhibitor reduced atherosclerotic associated with cardiovascular events only moderately (≈15%). Treatment targeting immune cells required a detailed understanding of the functional diversity of immune cells involved in atherosclerosis.

NFκB (RELA proto-oncogene, NF-kB subunit) and AKT (AKT serine/threonine kinase 1) are important transcription factors in regulating inflammation. For instance, oxidized LDLs (oxLDLs) or LPS triggers vascular inflammation leading to atherosclerosis through activated NFκB. The study suggests that increased oxLDLs lead to sustained activation of the scavenger receptor LOX-1 and, subsequently, to NFkB activation. [20]. Inflammation leads to increased proinflammatory cytokine levels, breaking the balance between pro- and anti-inflammatory cytokines. Therefore, recovering the anti-inflammatory balance might have protective effects against atherosclerosis [21].

Moreover, the AKT pathway participates in the survival, proliferation, and migration of macrophages, which may impact the development of atherosclerosis. Inhibition of AKT signaling in macrophages, which disrupts mTORC2 assembly, significantly decreases the proliferation of macrophages with the suppression of atherosclerosis [22]. Thus, the understanding of AKT signaling may have further learned in atherosclerosis, and AKT inhibitors might be drugs to treat patients.

Recent advances in the single-cell analysis provided an opportunity to study a variety of immune cells and the disease-related pathway associated with atherosclerosis, which can facilitate the identification of potential therapeutic targets [23–26]. In the present work, we reanalysis previously public database, patients [27] and mouse models of atherosclerosis [28,29].

## Materials and methods

### Patients

Forty-six patients undergoing carotid endarterectomy at Mount Sinai Hospital participated in an ongoing clinical study (data from *Prof*. Chiara Giannarelli’s lab). Peripheral blood and atherosclerotic samples were obtained from the paired patient [27]. Symptomatic (SYM) patients were defined as having had an event (e.g., stroke, transients ischemic attack within 6 months) ipsilateral to the collected plaque according to validated criteria [28,29]. Asymptomatic (ASYM) patients had no events within 6 months.

### Mice

Low-density lipoprotein receptor-deficiency (*Ldlr^−/−^*) mice were bred and housed under specific pathogen-free conditions. Six- to eight-week-old male mice were placed on a normal chow diet (*n*=9) or a high-fat diet (1.25% cholesterol) for 11 (*n*=8) and 20 weeks (*n*=7), while 8-week-old female Apo-lipoprotein E-deficiency (*Apoe^−/−^*) mice (*n*=10) were fed a chow diet or high-fat diet for 12 weeks. Aorta from the animals was pooled to generate samples for cell sorting of viable CD45^+^ cells and processed for single-cell RNA-sequencing [25]. All mice are on a C57BL/6 genetic background and housed under specific-pathogen-free conditions at the animal facilities (Model Animal Research Center of Nanjing University or West China Hospital of Sichuan University). All experiments were performed mice and approved by the Institutional Animal Care and Use Committee of Nanjing University (Animal Protocol MARC AP-FXY05). At the end of the experiments, mice were sacrificed by cervical dislocation, and tissues were collected. No other sedatives or anesthetics were used during the experiment.

### Oil Red O staining

Aortas were harvested and stained for atherosclerotic lesions using Oil Red O. Hearts were fixed in 10% buffered formalin, grossly cut through the ventricles parallel to the atria, and frozen in OCT (Tissue-Tek). Every other 10-micron section was placed on glass slides, stained with 0.2% Oil Red O (Sigma) in isopropanol for working solution, and counterstained with 2% hematoxylin.

### Real-time quantification PCR

Total RNA was extracted from tissue with TRNzol Reagent (TIANGEN, Catalog: DP405), according to the manufacturer’s instruction. Complementary DNA (cDNA) was synthesized with PrimeScript™ RT reagent kit with gDNA Eraser (Perfect Real Time) (Takara, Catalog: RR047A). Gene expressions were measured by the StepOne Plus Real-Time PCR system (Applied Biosystems) with SYBR qPCR kit (Takara, Catalog: RR820L). *Gapdh* was used as an internal control.

**Table:** 

Gene	Forward	Reverse
*Gapdh*	AACGGGAAGCCCATCACC	CAGCCTTGGCAGCACCAG
*Ifng*	GACAATCAGGCCATCAGCAACA	AACAGCTGGTGGACCACTCG
*Il10*	TGAATTCCCTGGGTGAGAAG	TGGCCTTGTAGACACCTTGG
*Il1b*	TACAGGCTCCGAGATGAACA	AGGCCACAGGTATTTTGTCG
*Il6*	GATGGATGCTACCAAACTGGA	CTCTGAAGGACTCTGGCTTTG
*Tnf*	GAACTGGCAGAAGAGGCACT	GAGGCCATTTGGGAACTTCT

### Western blot

Mice tissue and cells line (Jurkat & RAW264.7, ordered by ProCell Ltd.) were lysed by RadioImmunoPrecipitation Assay (RIPA) buffer (50 mM Tris-HCl, 150 mM NaCl, 1 mM EDTA, 1% NP-40, 0.1% SDS, 1 mM NaVO_4_, 1 mM NaF. Finally, pH 7.4). Protein quantification was performed using BCA Protein Assay Reagent (Thermo Fisher Scientific). Equal amounts of protein per sample (30 μg/ml) were loaded onto SDS-PAGE and transferred to PVDF membranes (Millipore). After blocking in TBST containing 5% BSA, membranes were incubated with primary antibodies at 4°C overnight. Membranes were subsequently incubated with HRP-conjugated goat antirabbit or goat antimouse secondary antibodies (1:10000 dilution). The protein bands were visualized and quantified using a chemiluminescence method (ECL System; Tanon).

Antibody information: Phospho-Akt (Ser473) (D9E) XP® Rabbit mAb (CST, #4060); Phospho-NF-κB p65 (Ser536) (93H1) Rabbit mAb (CST, #3033); Phospho-Stat1 (Tyr701) (58D6) Rabbit mAb (CST, #9167); GAPDH (D16H11) XP® Rabbit mAb (CST, #5174).

All of uncropped images of the western blots presented in Supplementary Figure S5A–G.

### Immunohistochemical

Paraffin-embedded tissue sections were stained by primary antibody and refer to the manual of UltraSensitive™ SP (mouse/rabbit) IHC Kit (MXB Biotechnologies, Catalog: KIT-9710) and DAB Signaling Amplification Kit (MXB Biotechnologies, Catalog: MAX007TM). Antibody: Phospho-Stat1 (Tyr701) (58D6) Rabbit mAb (CST, #9167).

### Volcano plot

DEGs from patients and mice were performed using http://www.ehbio.com/ImageGP/ analysis (parameter: gene symbol; log2 fold-change; *P*-value, and label).

### Gene Ontology pathway

DEGs from patients and mice were performed using http://geneontology.org/ analysis (analyze type: biological process).

Gene Ontology (GO) Enrichment plot analysis. Biological process data from GO analysis enriched and present GO Enrichment plot using http://www.ehbio.com/ImageGP/index.php/Home/Index/GOenrichmentplot.html (parameter: sample; generatio; *P*-value; count; description).

Protein–protein interaction by WGCNA modules.

Referred to the manual of STITCH website (http://stitch.embl.de/).

### Cytometry by time-of-flight data

Cytometry by time-of-flight (CyTOF) data were acquired with a CyTOF2 system using a SuperSampler fluidics system at an event rate of <400 events per second and normalized with Helios normalizer software. Data were uploaded to Cytobank (https://mtsinai.cytobank.org; Cytobank, 7.0) for analysis and visualization [27]. 41222 plaque cells with an average of 2748 cells per sample were analyzed. *P*-values were calculated using a paired, two-sided Student’s *t*-test using FDR correction by the Benjamini–Hochberg method. Fold-change and *P-*values were visualized using a volcano plot. Statistical analysis of cell population frequencies and marker expression using the multiple *t*-test with FDR correction, one-way analysis of variance (ANOVA), two-tailed Student’s *t*-test [27].

### LPS-induced proinflammation model on Jarkat and RAW264.7 cell line

We evaluated the effect of inflammation conditions (detecting protein level of P-AKT; P-NFKBp65; P-STAT1). Cells were cultured in fit plates, then treated with LPS (100 ng/ml) for 0, 1, 2, 4, or 24 h, harvesting the cell for next experiments.

### Statistics

All results are expressed as the mean ± SEM unless otherwise specified. Results were analyzed by Student’s *t*-test or ANOVA. The unpaired two-tailed Student’s *t*-test and one- way ANOVA were used for comparisons of experimental groups. The *P*-value of less than 0.05 was considered statistically significant. Statistical analysis was performed using GraphPad Prism software.

## Result

### Immune cells are enriched into plaques and PBMC in patients and mice with atherosclerosis

**Figure 1:**
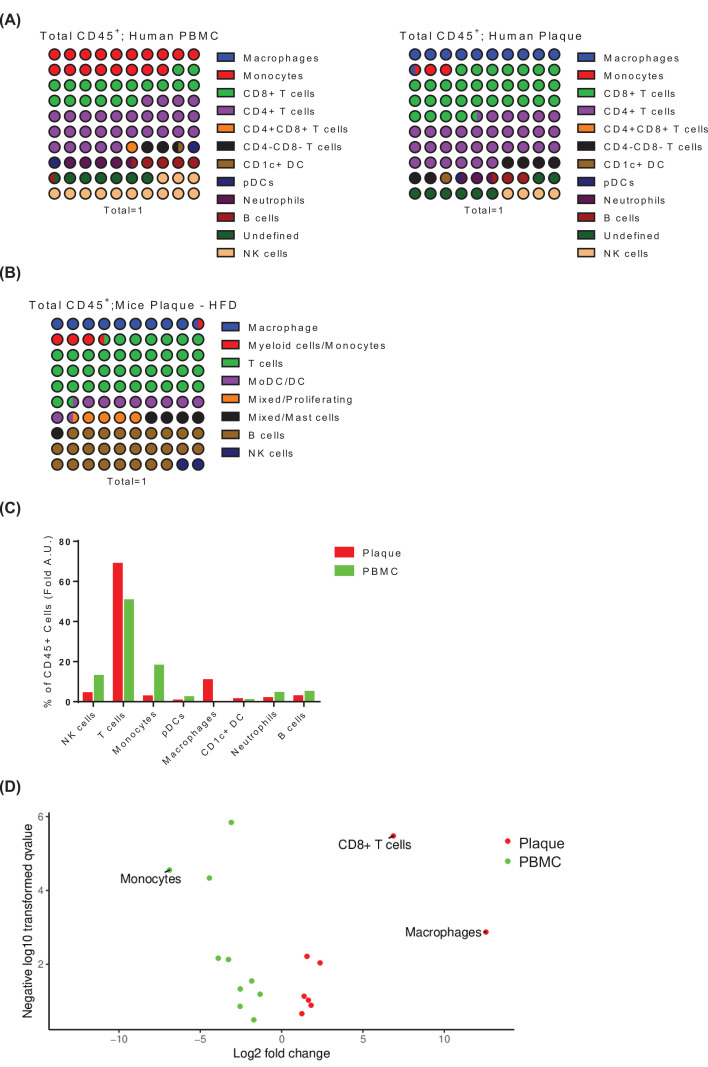
The landscape of immune cells in humans and mice with atherosclerosis (**A**) The panel of the relative frequency of immune cell types from human PBMC and plaque. DC (dendritic cell); pDC (plasmacytoid dendritic cell); NK cell (natural killer cell). (**B**) The panel of the relative frequency of immune cell types from *Apoe^−/−^* mice plaque. (**C**) The percentage of diverse immune cells in human PBMC and plaque. (**D**) Volcano plot of the cluster frequency in plaque and PBMC. *P*-values were calculated by *t*-test analysis.

To identify the types of resident immune cells in atherosclerotic plaques, we search and reanalysis the CyTOF and the scRNA-seq analysis in both patients and a mouse model of atherosclerosis from previously published works. In analyzing the database from the mass cytometry of atherosclerotic plaque and PBMC from patients, we found that all major immune cells (CD45^+^ as a marker) were identified in plaques and PBMC. T cells, especially the CD8^+^ T cells and macrophages were enriched in the human plaque (Figure 1A).

The median of ASYM and SYM groups was about 73-year-old (Supplementary Figure S1A). The lymphocytes of SYM slightly increased compared with ASYM (Supplementary Figure S1B). In the aspect of lipid/sterols, no significant difference was observed in plasma triglycerides level between ASYM and patients (Supplementary Figure S1C). Serum LDL (low-density lipoprotein) and HDL (high-density lipoprotein) levels, indicators of atherosclerosis [30], were not significantly different but the ratio of LDL/HDL increased in patients (Supplementary Figure S1D–F).

Moreover, similar results were observed in atherosclerotic plaque in mice. T cells and macrophages were enriched in plaque (Figure 1B). In contrast, PBMC contained more monocytes, NK cells, pDCs, and B cells (Figure 1C). It showed that T cells and macrophages were recruited into atherosclerotic plaques by the Volcano plot analysis (Figure 1D and Supplementary Table S2). These data indicated that immune cells recruitment to plaque in atherogenesis during the development of atherosclerosis, especially T cells and macrophage.

### DEGs of T cells and macrophages in patients and potential targets for drug development

To further identical key genes and signaling pathways in plaque and PBMC, we reanalysis the CITE-seq data from database. A total of 46 patients undergoing carotid endarterectomy were included and divided into two groups based on cardiovascular events (29 ASYM and 17 SYM) (Supplementary Table S1).

The immune cells in the plaque were mainly T cells and macrophages. We first analyzed the DEGs in T cells from patients (ASYM and SYM) (Supplementary Table S3), which showed that transcriptional markers associated with T-cell activation (*LYZ*, *NFATC2, TYROBP*) were highly expressed. In addition, cytokines and chemokines (*IFNG*, *CCL4*, *CXCR4*) were highly expressed in ASYM and SYM (Supplementary Figure S4A,C,D).

Meanwhile, macrophages analysis showed that cytotoxicity-related genes (*GZMA*, *GAMK*, *GIMAP7*) were highly expressed in SYM patients, while chemokines (*CXCL2*, *IL1B*) were highly expressed in ASYM patients (Supplementary Figure S4B). These data suggested that chemotaxis may be activated before symptoms emerged. The GO pathways analysis showed inflammatory functions, interferon/cytokines/chemokines signaling in T cells and macrophages (Supplementary Table S5). T cells up-regulated signaling associated with cell communication.

In PBMC, T cells were mostly in the resting phase, while in plaque, T cells showed varying degrees of activation, such as *STAT3*, *IFNGR1*, *HLA-B*, *CCL5*, *CXCR6, CCL4* (Supplementary Table S3). Signaling pathway analysis confirmed the presence of proinflammatory signaling (interferon-γ [IFN-γ] signaling) and T cells exhausted signaling in the plaques (Supplementary Table S5).

**Figure 2:**
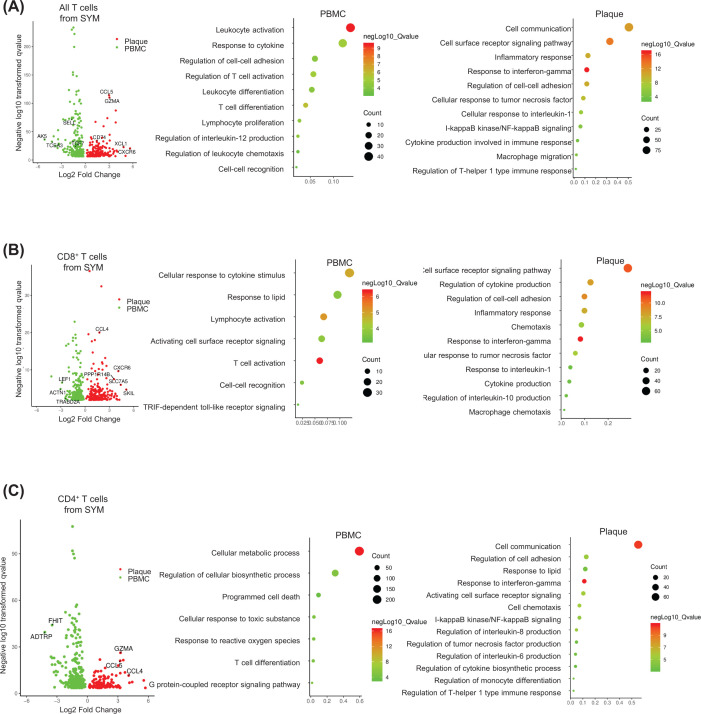
Gene expression of T cells in human with atherosclerosis (**A**) The volcano plot showed selected DEGs in human and GO pathway analysis. All T cells from SYM (*n*=17) plaque and PBMC. (**B**) The volcano plot showed selected DEGs in human and GO pathway analysis. CD4^+^ T cells from SYM (*n*=17) plaque and PBMC. (**C**) The volcano plot showed selected DEGs in human and GO pathway analysis. CD8^+^ T cells from SYM (*n*=17) plaque and PBMC.

Additionally, to further investigate the function of T cells in plaque or PBMC from SYM, we first focused on genes expressed in T cells at atherosclerotic plaques from SYM. Genes associated with cytotoxicity (*CXL1, GZMA*) and chemokines (*CCL5*, *CXCR6*) associated with cell communication and chemotaxis were highly expressed in plaques (Figure 2 and Supplementary Table S4). It suggested that T cells were not only activated and released chemokines to recruit immune cells but also performed killing function. Signaling GO pathways analysis up-regulated inflammatory functions and interferon/chemokines signaling in T cells (Supplementary Table S6), which indicated that above-target signaling pathways may be used in patients with atherosclerosis.

We list the common genes and potential genes from treatment of atherosclerosis (Table 1). Chemokine signaling (*CCR9*, *CXCR4*), JAK/STAT signaling (*IFNG*), cell communication (*TYROPB*), and Toll-like receptor (*S100A8*) are involved in atherosclerosis. Studies on the role of *TCF7* and *XCL1* genes in atherosclerosis have not been widely reported, but these genes are highly expressed in immune cells of patients and mice with atherosclerosis, which may become potential therapeutic targets.

**Table 1:** Potential therapeutic targets for atherosclerosis revealed by sequencing

Gene	Gene product	GWAS description	Signaling pathway	Related with atherosclerosis	Ref. (PMID)
TCF7	Transcription factor 7	Genetic risk and a primary role for cell-mediated immune mechanisms in multiple sclerosis	Wnt/β-catenin signaling	N/A	21833088
CCR9	C-C motif chemokine receptor 9	Multiple common variants for celiac disease influencing immune gene expression	Chemokine signaling	CCR9-CCL25 axis aggravate atherogenesis	20190752; 20504763
SOX4	SRY-box transcription factor 4	Hypertrophy-associated polymorphisms ascertained in a founder cohort applied to heart failure risk and mortality	NFκB; PI3K/AKT signaling	N/A	21348951; 28627651; 31109400
CXCR4	C-X-C chemokine receptor type 4	Meta-analysis of genome scans and replication identify CD6, IRF8 and TNFRSF1A as new multiple sclerosis susceptibility loci	C-C chemokine binding	CXCL12-CXCR4 axis aggravate atherogenesis	19525953; 21873635; 31145896
IFNG	Interferon-γ	Host–microbe interactions have shaped the genetic architecture of inflammatory bowel disease	JAK-STAT signaling	T-Bet/RUNX3-IFNG axis aggravate atherogenesis	23128233; 22504299; 23629966
FAS	Tumor necrosis factor receptor superfamily member 6	Association of IFIH1 and other autoimmunity risk alleles with selective IgA deficiency	Fas/FasL signaling	FAS-FASL axis attenuate atherogenesis	20694011; 11096076; 17259598
TYROBP	TYRO protein tyrosine kinase-binding protein	N/A	Cell–cell communication; DAP12 signaling	TREM1-DAP12 axis aggravate atherogenesis	9490415; 30070336
S100A8	Cystic fibrosis antigen	N/A	Toll-like receptor signaling	S100A8/S100A9-TLR4- NFκB aggravate atherogenesis	22489132; 31794767; 22095980
XCL1	X-C motif chemokine ligand 1	N/A	CCR chemokine receptor binding; IFN-γ	N/A	21873635
TCEA3	Transcription elongation factor A3	Genetic association study of QT interval highlights the role for calcium signaling pathways in myocardial repolarization	TGF-β; JNK signaling	N/A	23357533
LEF1 (TCF10)	Lymphoid enhancer-binding factor 1	A genome-wide association study identified AFF1 as a susceptibility locus for systemic lupus erythematosus	Wnt/β-catenin signaling	LEF1-AS1-PTEN axis aggravate atherogenesis	22291604; 31016789
ADTRP	Fatty acid esters of hydroxy fatty acids hydrolase	Evidence for gene–environment interaction in a genome-wide study of nonsyndromic cleft palate	PI3K/AKT signaling	N/A	28341552

### DEGs and GO pathway of T cells and macrophages in mice

To investigate and simulate human atherosclerosis, *Ldlr^−/−^* and *Apoe^−/−^* mice were commonly used in cardiovascular diseases studies. Total CD45^+^ leukocytes were isolated from the aortas for scRNA-sequencing. In mouse plaque cell, 13 distinct cell clusters were found to be correlated with *Ldlr^−/−^* mice plaque immune cell population by USBC (unsupervised seurat-based clustering); eight distinct cell clusters were found in *Apoe^−/−^* mice plaque (Figure 1B).

**Figure 3:**
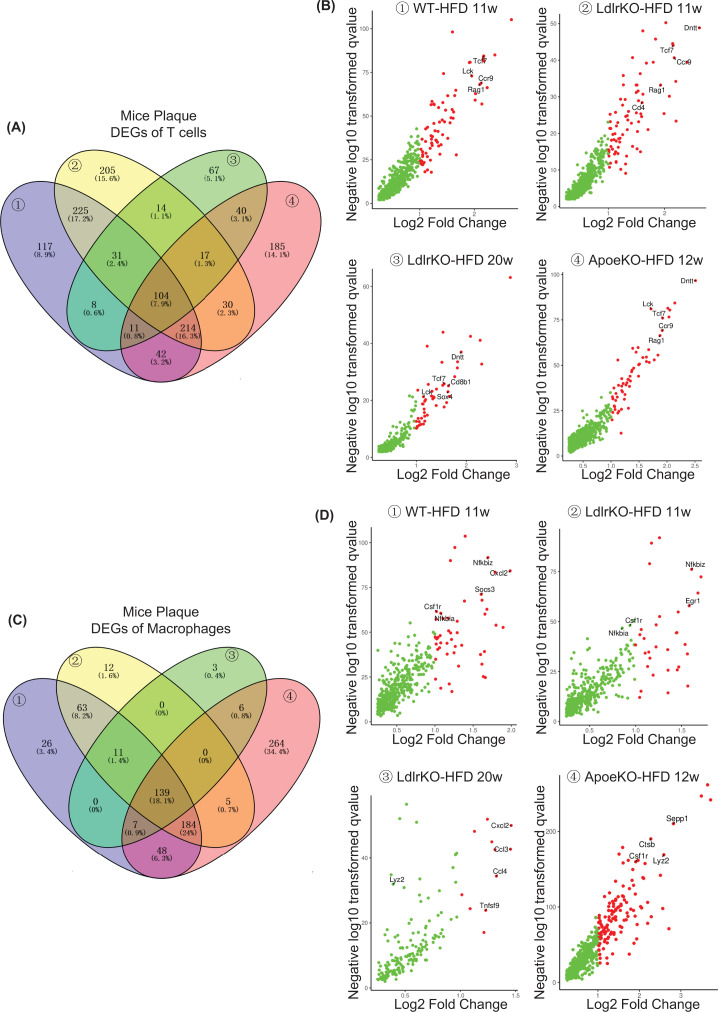
Gene expression of T cells and macrophages in mice with atherosclerosis (**A**) Venny picture showed DEG of T cells in atherosclerotic plaque among wild-type (WT) mice, *Ldlr*-deficiency mice and *Apoe*-deficiency mice with a high-fat diet. (**B**) The volcano plot showed selected DEGs in diverse mice plaque. (**C**) Venny picture showed DEG of macrophages in atherosclerotic plaque among WT mice, *Ldlr*-deficiency mice and *Apoe*-deficiency mice with a high-fat diet. (**D**) The volcano plot showed selected DEGs in diverse mice plaque.

Immune cells are present in the aorta of both healthy and patients. *Tcf7*, *Lck*, *Ccr9*, *Rag1*, *Dntt*, and *Sox4* were enriched in T cells (Figure 3A,B), which participate in immune response and chemokine signaling. *Nfkbia*, *Nfkbiz*, *Cxcl2*, *Ccl3*, *Ccl4*, *Csf1r*, *Egr1*, and *Sepp1* were enriched in macrophages (Figure 3C,D), which participate in inflammation and chemokine recruitment signaling. *Nkg7*, *Gzmk*, *Ifng*, and *Itgb1* were enriched in CD8^+^ T cells (Supplementary Figure S2A,B). *Cd79a*, *Ebf1, Ly6d*, and *Mzb1* were enriched in B cells (Supplementary Figure S2C,D). *Ncr1*, *Nkg7*, and *Irf8* were enriched in NK cells (Supplementary Figure S2E,F).

Moreover, monocytes (*Cstb*, *Psap*, *Msrb1*, *Tgfbi*), mast cells (*Il1rl1*, *Tnfrsf18*, *Nfkb1*, *Rora*, *Csf2*), and Neutrophils (*Retnlg*, *Ngp*, *Lcn2*, *Mmp9*) were identified in WT mice plaque with HFD (Supplementary Figure S3A). Monocytes (*Igals3*, *Plin2*, *Thbs1*, *Osm*) and Granulocytes (*Arg2*, *Mmp9*, *Nlrp3*, *Csf3r*) were identified in *Ldlr*-deficiency mice with HFD (Supplementary Figure S3B). Monocyte-driven dendric cells (*Napsa*, *Lsp1*, *Il1b*, *Fabp5*) were identified in *Ldlr*-deficiency mice with 20 weeks HFD (Supplementary Figure S3C). Monocyte-driven dendric cells (*Cst3*, *Cd209a*, *Lgals3*), monocytes (*Msrb1*, *Cebpb*, *Cstb*), and mast cells (*Furin*, *Csf2*, *Nfkb1*, *Il1rl1*) were identified in *Apoe*-deficiency mice with HFD (Supplementary Figure S3D).

Those DEGs in diverse immune cells implied that the immune system was in homeostasis and that the most abundant types of immune cells, such as T cells and macrophages, have effect on atherogenesis.

It was noteworthy that *Tcf7* and* Ccr9* were highly expressed in T cells (Figure 2B), while *Nfkb*, *Csf1r*, *Tnfsf9*, and *Ccl2* were highly expressed in macrophages (Figure 2D) in atherosclerotic plaque. NFκB signaling associated with proinflammatory and C-C chemokine signaling associated with cell communication/recruitment was activated in immune cells. These data could suggest that T cells recruited macrophages through the secretion of chemokines resulting in the aggravation of inflammation in atherogenesis. The significance of these genes in patients and mice with atherosclerosis provided an opportunity to study the mechanism and developed and potential drug.

**TCF7 and XCL1 were involved in AKT and NF**κ**B signaling pathways.**

**Figure 4:**
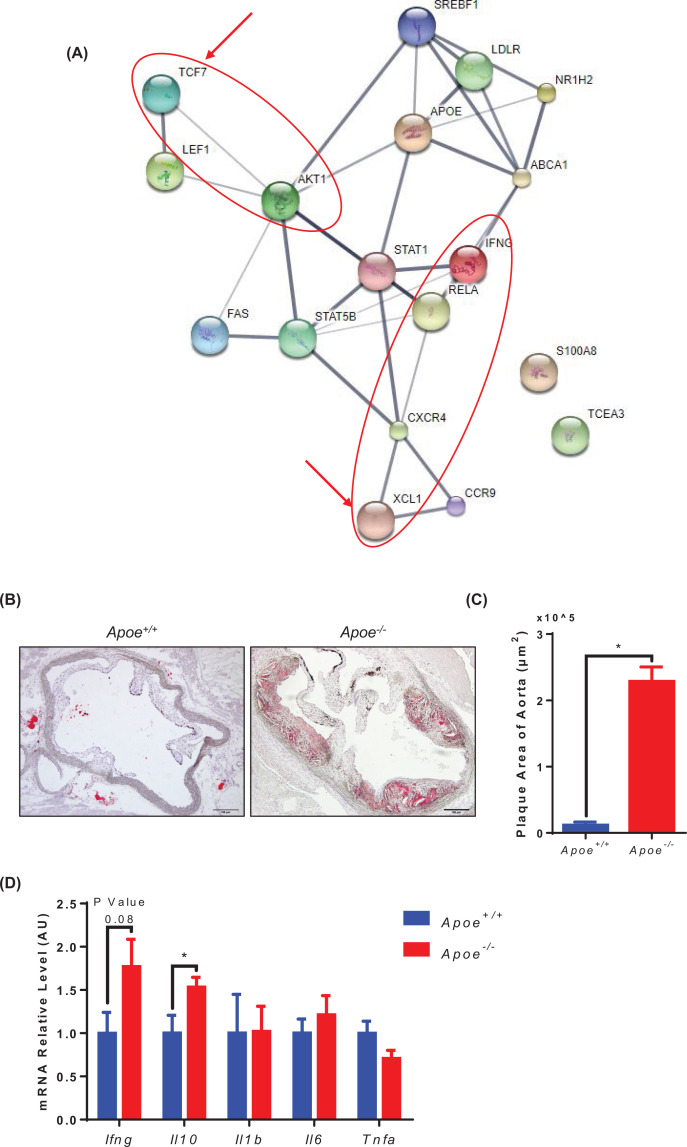
Core modules of network regulation of TCF7, XCL1 (**A**) The network regulation panel showed the relevancies of TCF7, XCL1, and AKT, NFκB signaling using ‘http://stitch.embl.de/’ website. (**B**) Representative sections of mice aorta root and staining with Oil Red O are shown. (**C**) Quantitative analyses of lesion area of aorta root staining (*n*≥5; mean ± SEM, *P<0.05). (**D**) The mRNA expression of *Ifng*, *Il10*, *Il1b*, *Il6*, *Tnf* involved in inflammatory cytokine in mice spleen, normalized to *Gapdh* (*n*=6, mean ± SEM, *P<0.05).

To determine which signaling pathway the novel genes obtained from bioinformatic analysis were involved in, we analyzed the differential genes (Table 1) by protein–protein interaction in the most enriched WGCNA module. We found that TCF7 and XCL1 could be regulated by AKT1 and NFκB (Synonym: Rela), respectively (Figure 4A). *Apoe*-deficiency mice were commonly used to simulate human atherosclerosis. The Oil Red O staining sections of mouse aorta displayed that the plaques were more aggressive in *Apoe^−/−^* mice than those of control mice (Figure 4B). There was also a statistically significant difference (Figure 4C).

As the data showed, TCF7 and XCL1 were regulated by AKT and NFκB, respectively. Thus, we examined the gene expression of *Apoe^−/−^* and control mice. We found that *Ifng* and *Il10* were increased in atherosclerotic mice (Figure 4D).

Those data illustrated that atherosclerosis is associated with inflammation. Moreover, the proinflammatory transcription factor, AKT/NFκB, was able to regulate downstream genes including TCF7, XCL1, and IFNG.

**Activation of the AKT, NF**κ**B signaling pathway was found in T cell and macrophages after LPS stimuli to simulated a proinflammatory environment.**

**Figure 5:**
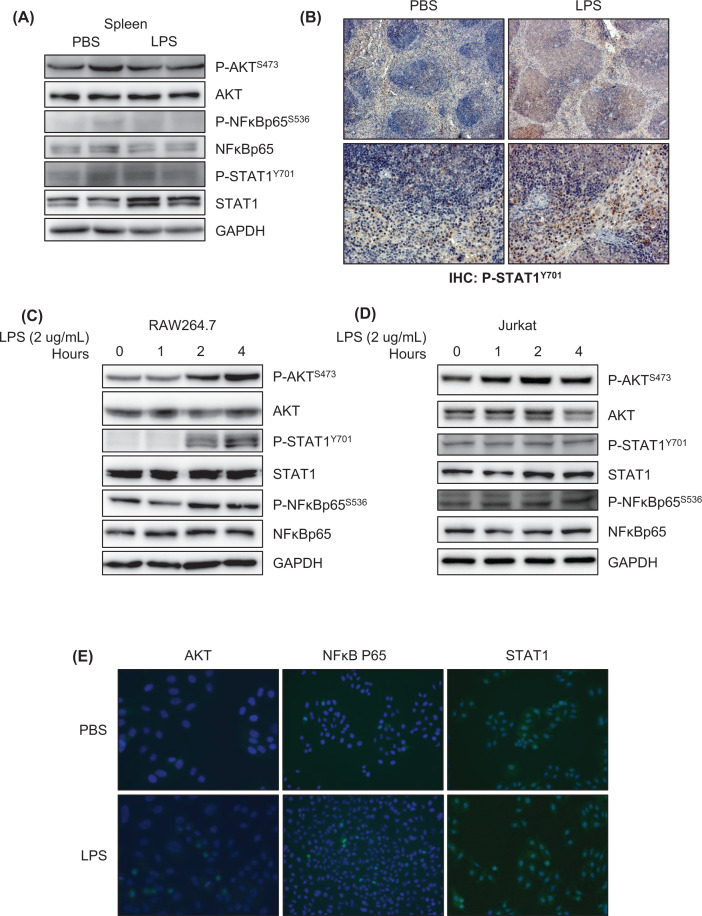
Activation of AKT/NFκB signaling in mice and cell lines under the inflammatory environment (**A**) Representative western blots analysis of P-AKT^S473^, AKT, P-NFκB^S536^, NFκB, P-STAT1^Y701^, STAT1, GAPDH protein involved in inflammation in mice spleen after LPS stimulating for 24 h. (**B**) A representative panel of immunohistochemical staining P-STAT1^Y701^ on the spleen section. (**C**) Representative western blots analysis of P-AKT^S473^, AKT, P-NFκB^S536^, NFκB, P-STAT1^Y701^, STAT1, GAPDH protein involved in inflammation in RAW264.7 macrophages cell line after 2 μg/mL LPS stimulating *in vitro*. (**D**) Representative western blots analysis of P-AKT^S473^, AKT, P-NFκB^S536^, NFκB, P-STAT1^Y701^, STAT1, GAPDH protein involved in inflammation in Jurkat T-cell line after 2 μg/mL LPS-stimulating *in vitro*. (**E**) Representative immunofluorescent analysis of AKT, NFκB, STAT1 protein involved in inflammation in cell line after 2 μg/mL LPS-stimulating *in vitro*.

In previous researches, LPS aggravated the atherogenesis in mice by increasing the proinflammatory environment [31,32]. Thus, we first examined the protein expression of AKT, NFκB, and STAT1. As we expected, the protein level of STAT1 increased in mice spleen after LPS stimulation (Figure 5A). In the meanwhile, similar conclusions were also displayed in the section of mice spleen. LPS-stimulated STAT1 phosphorylation level in the red pulp was highly expressed compared with control (Figure 5B).

Histological sections mainly divided the spleen into two parts. One is red pulp, enriching monocytes including macrophage. The other is white pulp, enriching T cells and B cells. To verify the expression and activation of the inflammatory transcription factor, we designed the LPS-induced proinflammation model on Jurkat (T cells) and RAW264.7 (macrophages) cell line *in vitro*. The phosphorylation of STAT1, AKT, and NFκB were elevated in a time-cross under LPS stimulation in macrophages (Figure 5C). However, LPS-induced inflammation was not different in T cells as significantly as in macrophages (Figure 5D). In the meantime, it showed the same result through the immunofluorescence method, in which LPS gave rise to an inflammatory microenvironment compared with the PBS group (Figure 5E).

To sum up, the proinflammatory environment did aggravate atherosclerosis. We demonstrated that the AKT1, NFκB, STAT1 signaling pathway was activated in immune cells by LPS stimulation *in vitro*. TCF7 and XCL1 were regulated by AKT1 and NFκB, respectively. Thus, those genes may be potential therapeutic targets via AKT/NFκB signaling pathway.

## Discussion

In the present study, we performed to reanalysis of single-cell sequencing data of atherosclerotic plaques and PBMC immune cells and found that their T cells and macrophages showed dysfunction. It has been a challenge to clarify the role of the immune system in atherogenesis. In Dennis Wolf’s research, immune components of plaque/PBMC have been inferred by RNA-seq analysis of atherosclerotic plaques [23]. The main components of immune cells are T cells and macrophages in atherosclerosis. However, RNA-sequencing data were limited to the average of gene expression patterns across the entire tissues. Thus, it was an approach to solve this problem by single-cell sequencing, which could detect the gene expression of one cell. In recent years, several bioinformatics methods have been proposed for the analysis and interpretation of scRNA-seq data [33].

Not only were plaque T cells primary present in plaques but they were more active and differentiated than PBMC in humans, similar results in mice. The subpopulations of activated T cells in plaques suggested that highly activated T cells may trigger chronic inflammation and release proinflammatory cytokines genes (*TNF, IL1B, IFNG*) and chemokines genes (*CCL2, CCL5, CXCL6*) to recruit immune cells to participate in atherogenesis [34]. Gene expression of plaque macrophages indicated a diverse function on single-cell transcription levels. Macrophage displayed activated and proinflammatory function (*IFNG*, *NF*κ*B*), meanwhile expressed genes involved in lipid metabolism (*APOE, ABCA1, ABCG1*), similar to foam cell functions. These data indicated the functional phenotype and underlying pathological mechanism of macrophages in the plaque.

The activated T cell in SYM plaques was significantly increased. In patients, the gene expression characteristics of all T-cell lineages were consistent with activation and differentiation. Nevertheless, it was most important to understand the changes in molecular mechanisms of T cells and macrophages in atherogenesis to accurately treat them. In our hypothesis, cell communication between T cells and macrophages could play a key role, suggesting that T cells drive inflammation and recruit macrophages to atherosclerotic plaques, thereby aggravated the progression of atherosclerosis. Considering that activated T cells could aggravate atherosclerosis, immunosuppressive therapy may partially alleviate atherosclerosis [35]. Similar to plaque T cells, macrophages were activated and proinflammatory in patients. In general, the specific functions of macrophages in atherosclerotic plaques depended on the regulation of T-cell ligand signaling. Those results implied a unique and highly co-ordinated innate and adaptive communications in atherosclerotic plaque.

*IL1B* and *IFNG* were highly expressed in macrophages of patients’ plaque, which were involved in IL-1 and IFN-γ signaling pathway. Moreover, we found that IL-1 and IFN-γ have activated proinflammatory not only in macrophages but also in T cells. This indicated that there was cross-talk between innate and adaptive immune responses in plaques. In a previous study, immunohistochemical analysis of plaque detected the high expression of several genes enriched in inflammatory macrophages such as TNF-α and TNFSF9 [25]. Thus, those results implied that IL-1, IFN-γ, and TNF signaling were highly activated in atherogenesis. It could inhibit proinflammatory signaling to treat patients.

To sum up, combined with single-cell analysis of atherosclerotic plaques and PBMC in patients or mice, cell communication between T cell and macrophages were proposed. The proinflammatory signaling was highly activated to recruit immune cells at the plaques to aggravate atherogenesis. In addition to the common inflammatory signaling pathway, there are still several genes that are highly expressed in plaque but not widely studied in patients. *TCF7* and *XCL1* genes may be a potential and novel target for therapy through AKT/NFκB pathway.

## Perspectives

- Atherosclerosis is one of the leading cardiovascular disease, with high global mortality rates. Nevertheless, the underlying mechanism remains largely unknown.
- We identify two novel genes *TCF7* and *XCL1* derived from public single-cell-sequencing database of patients with atherosclerosis. These genes participate in proinflammatory signaling (regulated by NFκB/AKT).
- Further studies of atherogenesis mechanisms by taking advantage of single-cell-sequencing technologies provide novel target genes associated with inflammation into the pathology of atherosclerosis and it may be new drug targets for therapy.

## Supplementary Material

Supplementary Figures S1-S5

Supplementary Tables S1-S6

## Data Availability

The data that support the findings of the present study are available on request from the authors at link below. The data discussed in this publication are from Prof. Chiara Giannarelli’s lab (https://figshare.com/s/c00d88b1b25ef0c5c788; DOI: 10.6084/m9.figshare.9206387) and GitHub with links to interactive Jupiter notebooks (https://zenodo.org/record/3361716; DOI: 10.5281/zenodo.3361716) [27]. The data discussed in this publication are from Prof. Alma Zernecke’s lab (Accession Number: GSE97310) [25].

## Abbreviations

ANOVA: analysis of variance
Apoe−/−: apo-lipoprotein E-deficiency
ASYM: asymptomatic
cDNA: complementary DNA
CyTOF: cytometry by time-of-flight
DC: dendritic Cell
DEG: differentially expressed gene
HDL: high-density lipoprotein
HFD: high-fat-diet
IFN: interferon
IL-1β: interleukin-1β
LDL: low-density lipoprotein
Ldlr−/−: low-density lipoprotein receptor-deficiency
NK: natural killer
oxLDL: oxidized LDL
PBMC: peripheral blood mononuclear cell
pDC: plasmacytoid dendritic cell
RIPA: radio-immunoprecipitation assay
SYM: symptomatic
WT: wild-type

## References

[1] Libby P. (2002) Inflammation in atherosclerosis. Nature 420, 868–874 10.1038/nature01323 12490960

[2] Ma Z., Sheng N., Liu X., Su Y., Zhou Y., Sun Y. et al. (2020) Knockout of Stat5 in T cells ameliorates high cholesterol and high fat diet-induced hypercholesterolemia by influencing cholesterol metabolism in the liver. Cell Mol. Immunol. 10.1038/s41423-020-0389-8 32139883

[3] Wolf D., Zirlik A. and Ley K. (2015) Beyond vascular inflammation–recent advances in understanding atherosclerosis. Cell. Mol. Life Sci. 72, 3853–3869 10.1007/s00018-015-1971-6 26100516 PMC4577451

[4] Tabas I. and Lichtman A.H. (2017) Monocyte-macrophages and T cells in atherosclerosis. Immunity 47, 621–634 10.1016/j.immuni.2017.09.008 29045897 PMC5747297

[5] Libby P. (2017) Superficial erosion and the precision management of acute coronary syndromes: not one-size-fits-all. Eur. Heart J. 38, 801–803 10.1093/eurheartj/ehw599 28053187

[6] Finn A.V., Nakano M., Narula J., Kolodgie F.D. and Virmani R. (2010) Concept of vulnerable/unstable plaque. Arterioscler. Thromb. Vasc. Biol. 30, 1282–1292 10.1161/ATVBAHA.108.179739 20554950

[7] Moreno P.R., Falk E., Palacios I.F., Newell J.B., Fuster V. and Fallon J.T. (1994) Macrophage infiltration in acute coronary syndromes. Implications for plaque rupture. Circulation 90, 775–778 10.1161/01.CIR.90.2.775 8044947

[8] Cochain C. and Zernecke A. (2016) Protective and pathogenic roles of CD8(+) T cells in atherosclerosis. Basic Res. Cardiol. 111, 71 10.1007/s00395-016-0589-7 27783202

[9] Su Y., Zhang W., Patro C.P.K., Zhao J., Mu T., Ma Z. et al. (2020) STAT3 regulates mouse neural progenitor proliferation and differentiation by promoting mitochondrial metabolism. Front. Cell Dev. Biol. 8, 362 10.3389/fcell.2020.00362 32509786 PMC7248371

[10] Sheng N., Ma Z., Zhou Y., Xu J., Gao Y. and Fu X.Y. (2020) Cholesterol 25-hydroxylase protects against experimental colitis in mice by modulating epithelial gut barrier function. Sci. Rep. 10, 14246 10.1038/s41598-020-71198-1 32859970 PMC7455728

[11] Dumitriu I.E., Baruah P., Finlayson C.J., Loftus I.M., Antunes R.F., Lim P. et al. (2012) High levels of costimulatory receptors OX40 and 4-1BB characterize CD4+CD28null T cells in patients with acute coronary syndrome. Circ. Res. 110, 857–869 10.1161/CIRCRESAHA.111.261933 22282196

[12] Methe H., Brunner S., Wiegand D., Nabauer M., Koglin J. and Edelman E.R. (2005) Enhanced T-helper-1 lymphocyte activation patterns in acute coronary syndromes. J. Am. Coll. Cardiol. 45, 1939–1945 10.1016/j.jacc.2005.03.040 15963390

[13] Ghattas A., Griffiths H.R., Devitt A., Lip G.Y. and Shantsila E. (2013) Monocytes in coronary artery disease and atherosclerosis: where are we now? J. Am. Coll. Cardiol. 62, 1541–1551 10.1016/j.jacc.2013.07.043 23973684

[14] Cochain C. and Zernecke A. (2017) Macrophages in vascular inflammation and atherosclerosis. Pflugers Archiv.: Eur. J. Physiol. 469, 485–499 10.1007/s00424-017-1941-y 28168325

[15] Tabas I. and Bornfeldt K.E. (2016) Macrophage phenotype and function in different stages of atherosclerosis. Circ. Res. 118, 653–667 10.1161/CIRCRESAHA.115.306256 26892964 PMC4762068

[16] Nahrendorf M. and Swirski F.K. (2016) Abandoning M1/M2 for a network model of macrophage function. Circ. Res. 119, 414–417 10.1161/CIRCRESAHA.116.309194 27458196 PMC4965179

[17] Agrawal S., Febbraio M., Podrez E., Cathcart M.K., Stark G.R. and Chisolm G.M. (2007) Signal transducer and activator of transcription 1 is required for optimal foam cell formation and atherosclerotic lesion development. Circulation 115, 2939–2947 10.1161/CIRCULATIONAHA.107.696922 17533179

[18] Ortiz-Munoz G., Martin-Ventura J.L., Hernandez-Vargas P., Mallavia B., Lopez-Parra V., Lopez-Franco O. et al. (2009) Suppressors of cytokine signaling modulate JAK/STAT-mediated cell responses during atherosclerosis. Arterioscler. Thromb. Vasc. Biol. 29, 525–531 10.1161/ATVBAHA.108.173781 19164812

[19] Gallego-Colon E., Daum A. and Yosefy C. (2020) Statins and PCSK9 inhibitors: a new lipid-lowering therapy. Eur. J. Pharmacol. 878, 173114 10.1016/j.ejphar.2020.173114 32302598

[20] Gliozzi M., Scicchitano M., Bosco F., Musolino V., Carresi C., Scarano F. et al. (2019) Modulation of nitric oxide synthases by oxidized LDLs: role in vascular inflammation and atherosclerosis development. Int. J. Mol. Sci. 20, 3294 PMC6651385 10.3390/ijms20133294 31277498 PMC6651385

[21] Yang Z., Kang L., Wang Y., Xiang J., Wu Q., Xu C. et al. (2019) Role of IL-37 in cardiovascular disease inflammation. Can. J. Cardiol. 35, 923–930 10.1016/j.cjca.2019.04.007 31292092

[22] Linton M.F., Moslehi J.J. and Babaev V.R. (2019) Akt signaling in macrophage polarization, survival, and atherosclerosis. Int. J. Mol. Sci. 20, 2703 PMC6600269 10.3390/ijms20112703 31159424 PMC6600269

[23] Winkels H., Ehinger E., Vassallo M., Buscher K., Dinh H.Q., Kobiyama K. et al. (2018) Atlas of the immune cell repertoire in mouse atherosclerosis defined by single-cell RNA-sequencing and mass cytometry. Circ. Res. 122, 1675–1688 10.1161/CIRCRESAHA.117.312513 29545366 PMC5993603

[24] Cole J.E., Park I., Ahern D.J., Kassiteridi C., Danso Abeam D., Goddard M.E. et al. (2018) Immune cell census in murine atherosclerosis: cytometry by time of flight illuminates vascular myeloid cell diversity. Cardiovasc. Res. 114, 1360–1371 10.1093/cvr/cvy109 29726984 PMC6054192

[25] Cochain C., Vafadarnejad E., Arampatzi P., Pelisek J., Winkels H., Ley K. et al. (2018) Single-cell RNA-seq reveals the transcriptional landscape and heterogeneity of aortic macrophages in murine atherosclerosis. Circ. Res. 122, 1661–1674 10.1161/CIRCRESAHA.117.312509 29545365

[26] Lin J.D., Nishi H., Poles J., Niu X., McCauley C., Rahman K. et al. (2019) Single-cell analysis of fate-mapped macrophages reveals heterogeneity, including stem-like properties, during atherosclerosis progression and regression. JCI Insight 4, e124574 PMC6478411 10.1172/jci.insight.124574 30830865 PMC6478411

[27] Fernandez D.M., Rahman A.H., Fernandez N.F., Chudnovskiy A., Amir E.D., Amadori L. et al. (2019) Single-cell immune landscape of human atherosclerotic plaques. Nat. Med. 25, 1576–1588 10.1038/s41591-019-0590-4 31591603 PMC7318784

[28] Herrmann J., Mannheim D., Wohlert C., Versari D., Meyer F.B., McConnell J.P. et al. (2009) Expression of lipoprotein-associated phospholipase A(2) in carotid artery plaques predicts long-term cardiac outcome. Eur. Heart J. 30, 2930–2938 10.1093/eurheartj/ehp309 19689974 PMC2785944

[29] Versari D., Herrmann J., Gossl M., Mannheim D., Sattler K., Meyer F.B. et al. (2006) Dysregulation of the ubiquitin-proteasome system in human carotid atherosclerosis. Arterioscler. Thromb. Vasc. Biol. 26, 2132–2139 10.1161/01.ATV.0000232501.08576.73 16778122

[30] Wang S. and Smith J.D. (2014) ABCA1 and nascent HDL biogenesis. Biofactors 40, 547–554 10.1002/biof.1187 25359426 PMC4294467

[31] Nogiec A., Bzowska M., Demczuk A., Varol C. and Guzik K. (2020) Phenotype and response to PAMPs of human monocyte-derived foam cells obtained by long-term culture in the presence of oxLDLs. Front. Immunol. 11, 1592 10.3389/fimmu.2020.01592 32849539 PMC7417357

[32] Nishikawa M., Kurano M., Ikeda H., Aoki J. and Yatomi Y. (2015) Lysophosphatidylserine has bilateral effects on macrophages in the pathogenesis of atherosclerosis. J. Atheroscler. Thromb. 22, 518–526 10.5551/jat.25650 25445889

[33] Chen G., Ning B. and Shi T. (2019) Single-cell RNA-seq technologies and related computational data analysis. Front. Genet. 10, 317 10.3389/fgene.2019.00317 31024627 PMC6460256

[34] Yi J.S., Cox M.A. and Zajac A.J. (2010) T-cell exhaustion: characteristics, causes and conversion. Immunology 129, 474–481 10.1111/j.1365-2567.2010.03255.x 20201977 PMC2842494

[35] Zhou M., Ren P., Zhang Y., Li S., Li M., Li P. et al. (2019) Shen-Yuan-Dan capsule attenuates atherosclerosis and foam cell formation by enhancing autophagy and inhibiting the PI3K/Akt/mTORC1 signaling pathway. Front. Pharmacol. 10, 603 10.3389/fphar.2019.00603 31214032 PMC6554665

