# Supplementary material for: TCF7 is highly expressed in immune cells on the atherosclerotic plaques, and regulating inflammatory signaling via NFκB/AKT/STAT1 signaling
Source: Biosci Rep. 2022 Jul 18;42(7):BSR20212064. doi: 10.1042/BSR20212064 (PMC9297684; doi:10.1042/BSR20212064)
Supplement: Supplementary Figures S1-S5 [file BSR-2021-2064_supp.pdf]

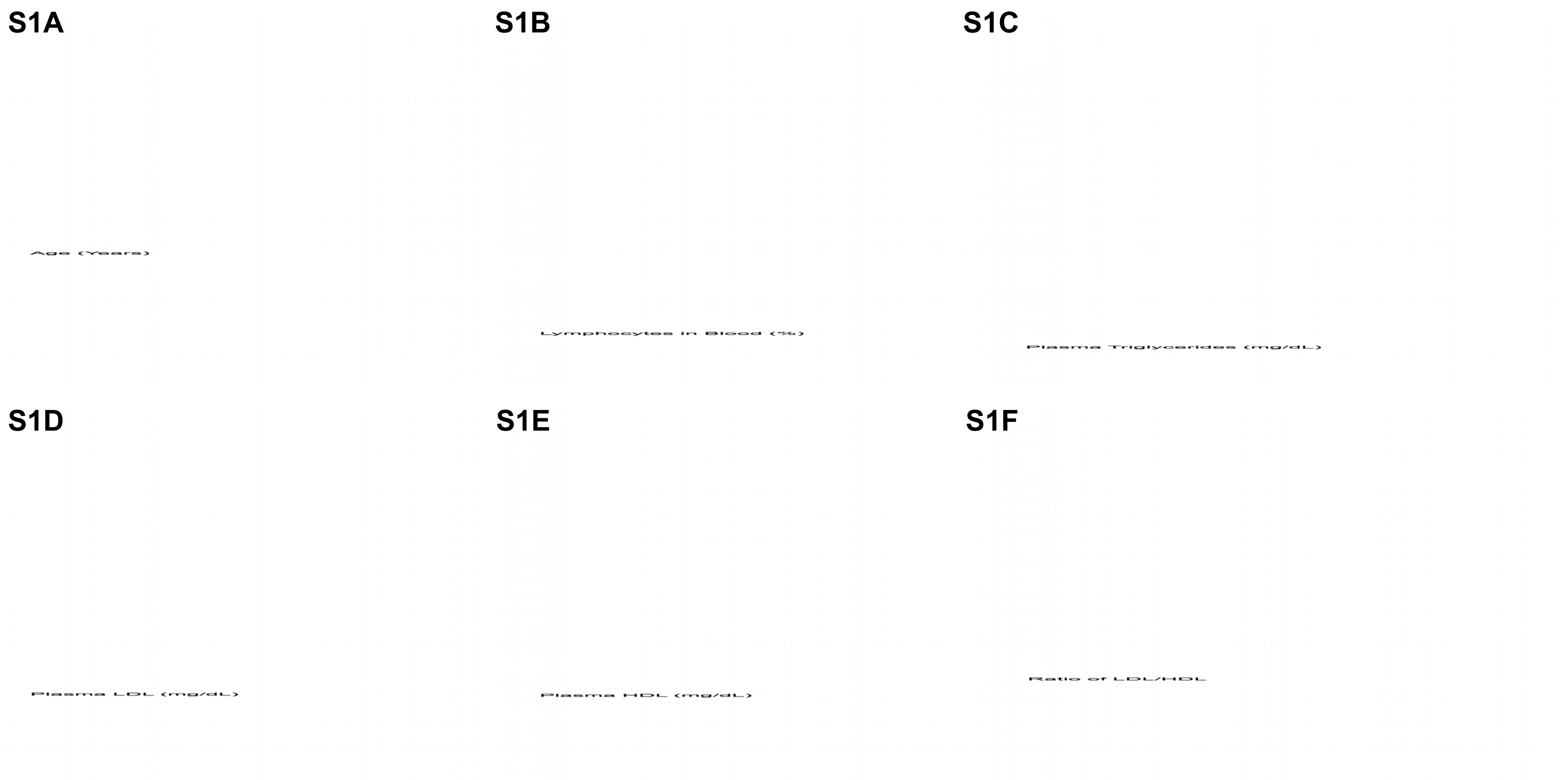

sFigure 1. Clinical characteristics of human.

(A) Statistics of human age; atherosclerosis patients without asymptomatic (stroke) within 6 months (as ASYM; n = 29); recent stroke patients (as SYM; n = 17).

(B) Lymphocytes in human blood by Blood Routine Tests. (ASYM n = 29; SYM n = 17).

(C) The concentration of triglycerides in human plasma (mg/dL). (ASYM n = 29; SYM n = 17).

(D) The concentration of Low-Density Lipoprotein in human plasma (mg/dL). (ASYM n = 29; SYM n = 17).

(E) The concentration of High-Density Lipoprotein in human plasma (mg/dL). (ASYM n = 29; SYM n = 17).

(F) The ratio of LDL/HDL in human. (ASYM n = 29; SYM n = 17).

S2A

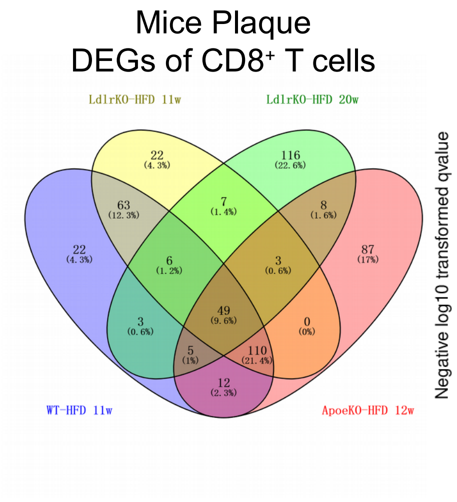

S2B

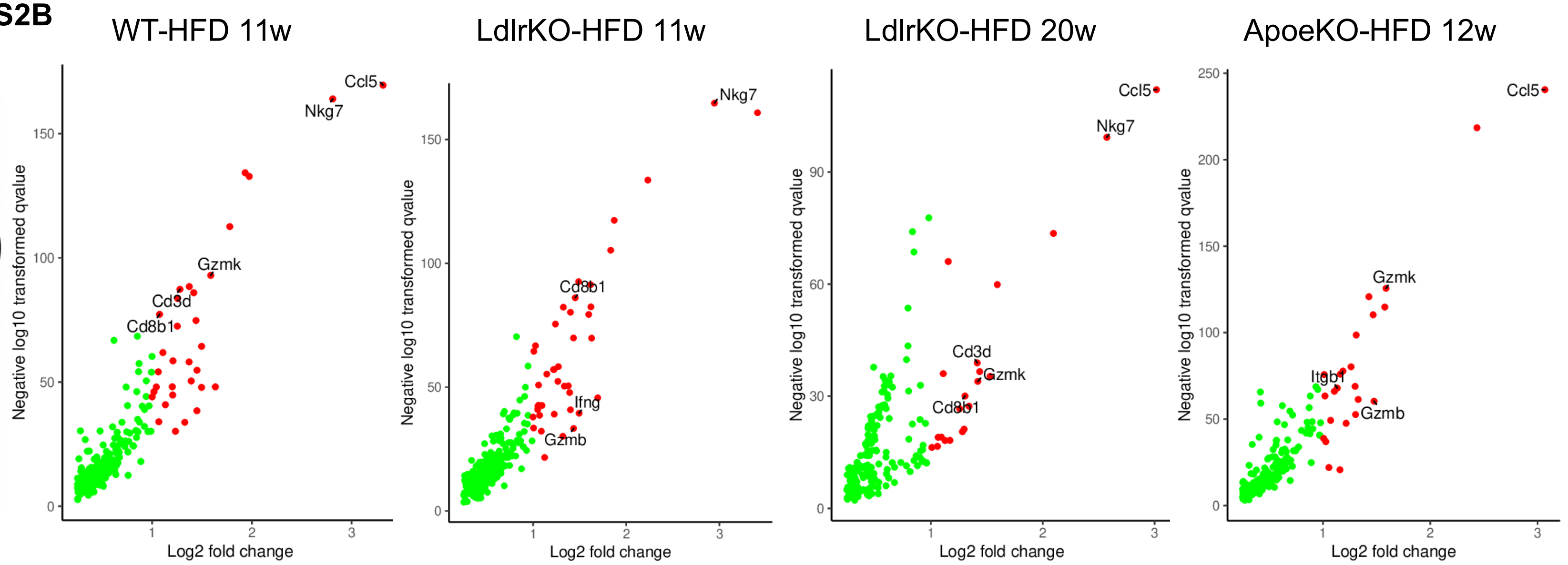

S2C

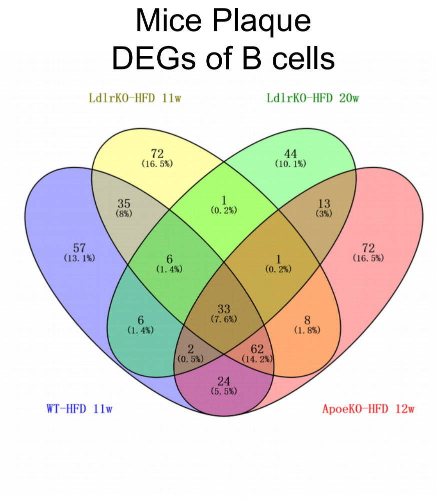

S2D

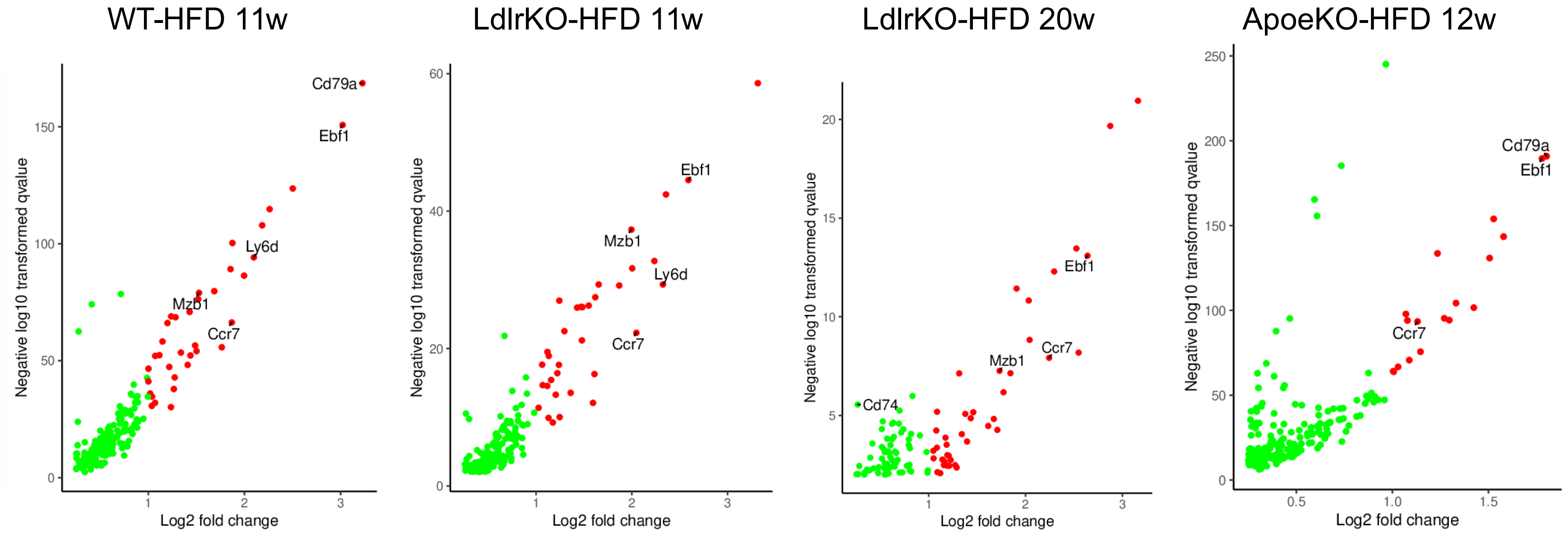

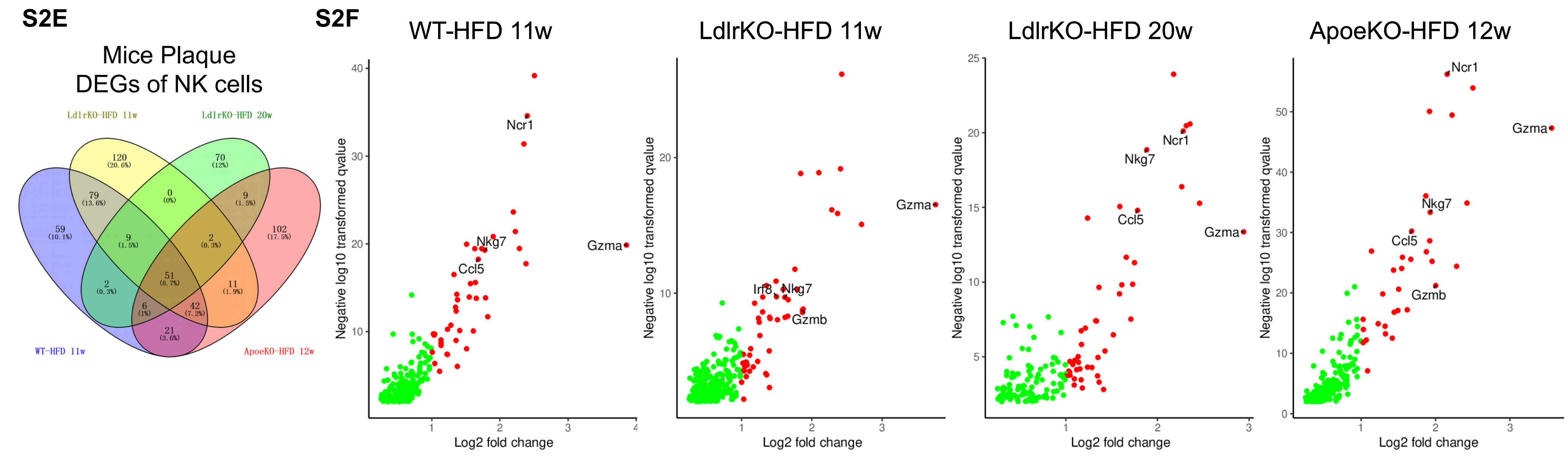

sFigure 2. gene expression of T cells in mice with atherosclerosis

(A) Venny picture showed DEG (Differentially Expressed Gene) of CD8⁺ T cells in atherosclerotic plaque among wild type mice, Ldlr-deficiency mice, Apoe-deficiency mice with high fat diet.

(B) Volcano plot showed selected differentially expressed genes in diverse mice plaque of sFigure 1A.

(C) Venny picture showed DEG of B cells in atherosclerotic plaque among wild type mice, Ldlr-deficiency mice, Apoe-deficiency mice with high fat diet.

(D) Volcano plot showed selected differentially expressed genes in diverse mice plaque of sFigure 1C.

(E) Venny picture showed DEG of NK cells in atherosclerotic plaque among wild type mice, Ldlr-deficiency mice, Apoe-deficiency mice with high fat diet.

(F) Volcano plot showed selected differentially expressed genes in diverse mice plaque of sFigure 1E.

S3A

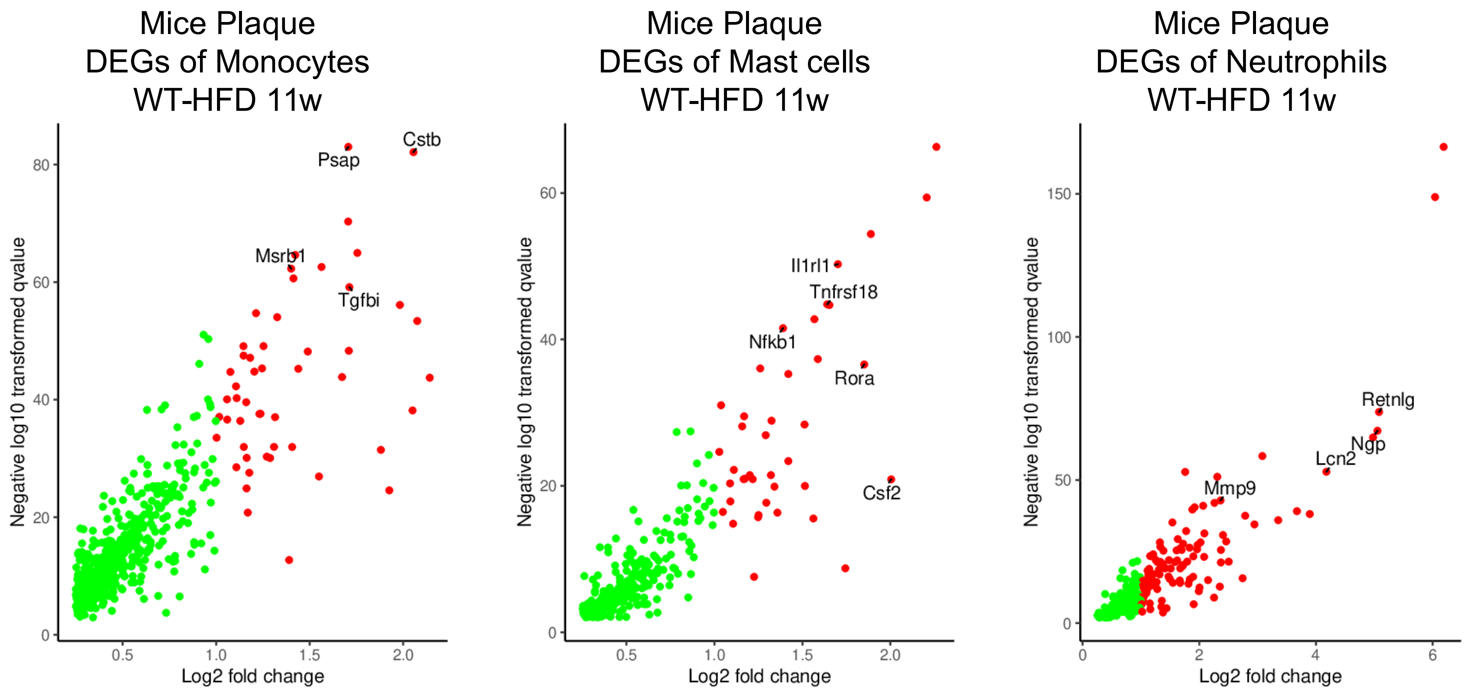
Mice Plaque
DEGs of Monocytes
WT-HFD 11w
Negative log10 transformed qvalue
Log2 fold change
Cstb
Psap
Msrb1
Tgfbi
Mice Plaque
DEGs of Mast cells
WT-HFD 11w
Negative log10 transformed qvalue
Log2 fold change
Il1rl1
Tnfrsf18
Nfkb1
Rora
Csf2
Mice Plaque
DEGs of Neutrophils
WT-HFD 11w
Negative log10 transformed qvalue
Log2 fold change
Retnlg
Ngp
Lcn2
Mmp9

S3B

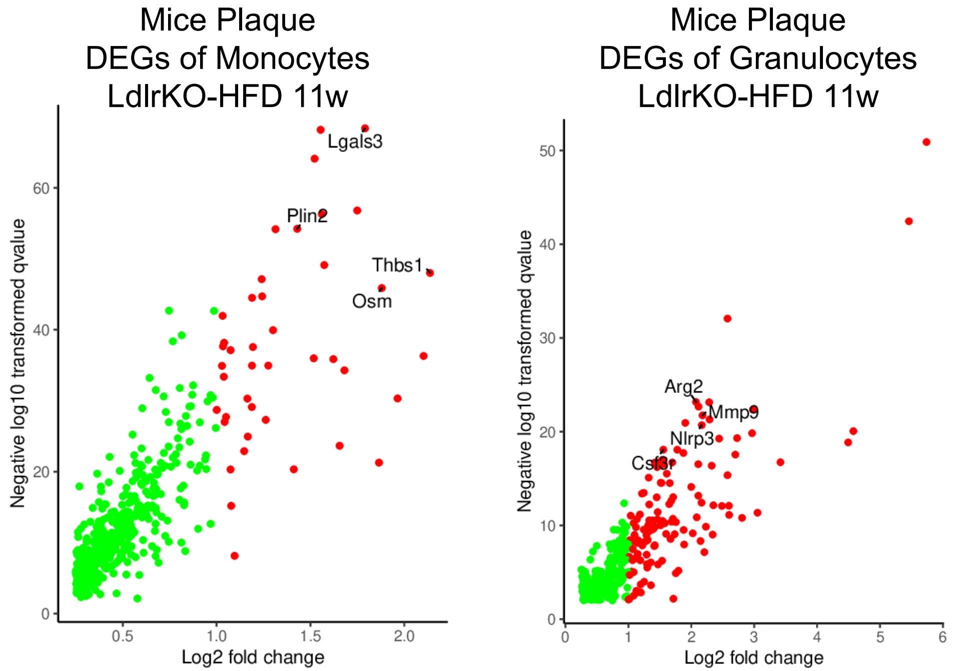
Mice Plaque
DEGs of Monocytes
LdlrKO-HFD 11w
Negative log10 transformed qvalue
Log2 fold change
Lgals3
Plin2
Thbs1
Osm
Mice Plaque
DEGs of Granulocytes
LdlrKO-HFD 11w
Negative log10 transformed qvalue
Log2 fold change
Arg2
Mmp9
Nlrp3
Csf3r

S3C

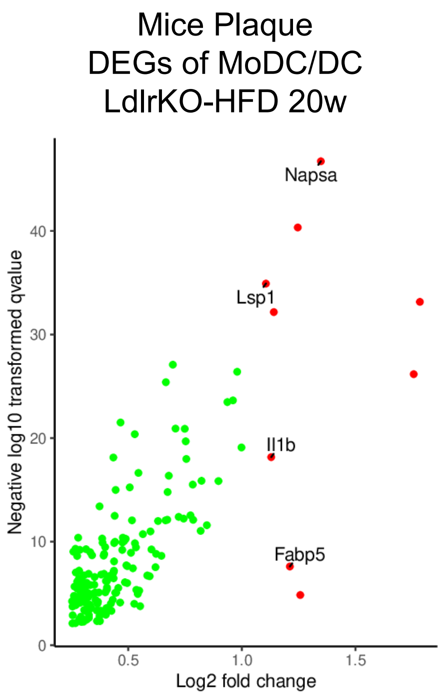

S3D

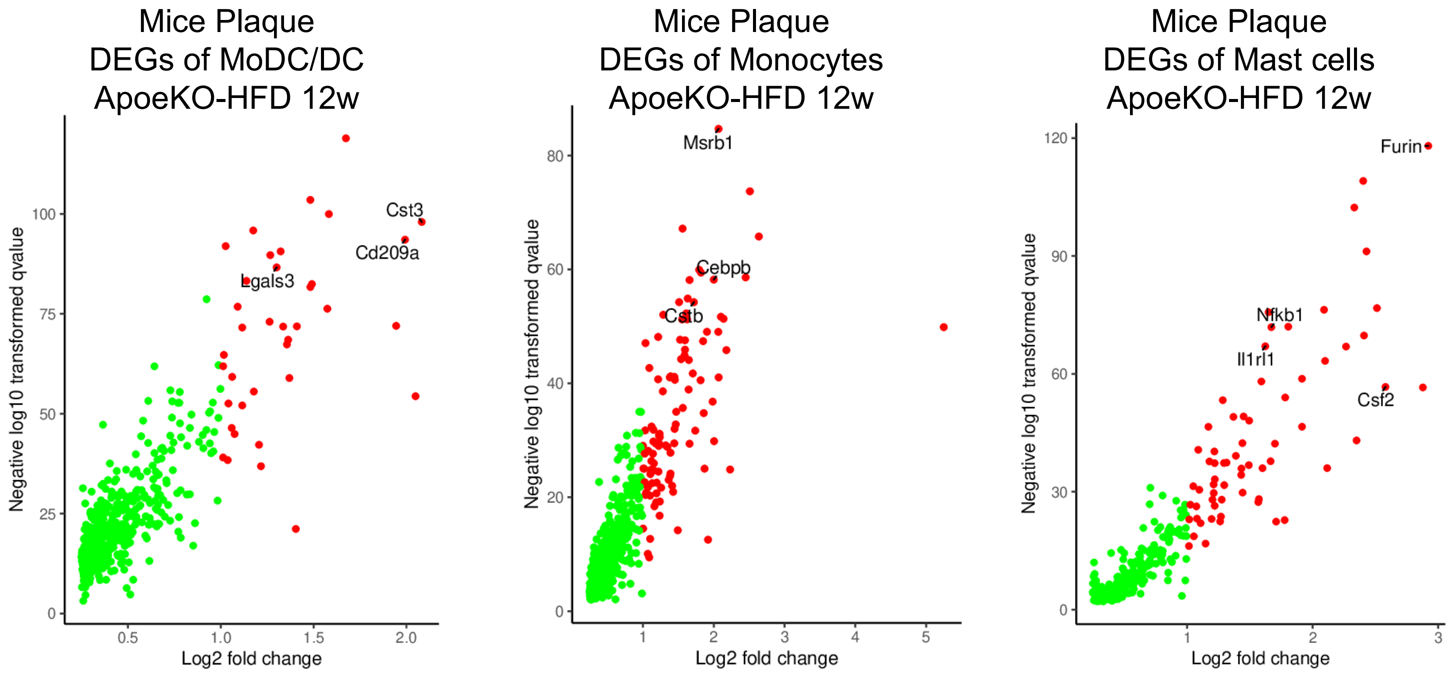

sFigure 3. gene expression of other immune cells in mice with atherosclerosis

(A) Volcano plot showed selected differentially expressed genes in WT mice with 11 weeks HFD. Monocytes, mast cells and Neutrophils.

(B) Volcano plot showed selected differentially expressed genes in *Ldlr*-deficiency mice with 11 weeks HFD. Monocytes and Granulocytes.

(C) Volcano plot showed selected differentially expressed genes in *Ldlr*-deficiency mice with 20 weeks HFD. Monocytes-driven dendric cells.

(D) Volcano plot showed selected differentially expressed genes in *Apoe*-deficiency mice with 12 weeks HFD. Monocytes-driven dendric cells, Monocytes and mast cells.

S4A

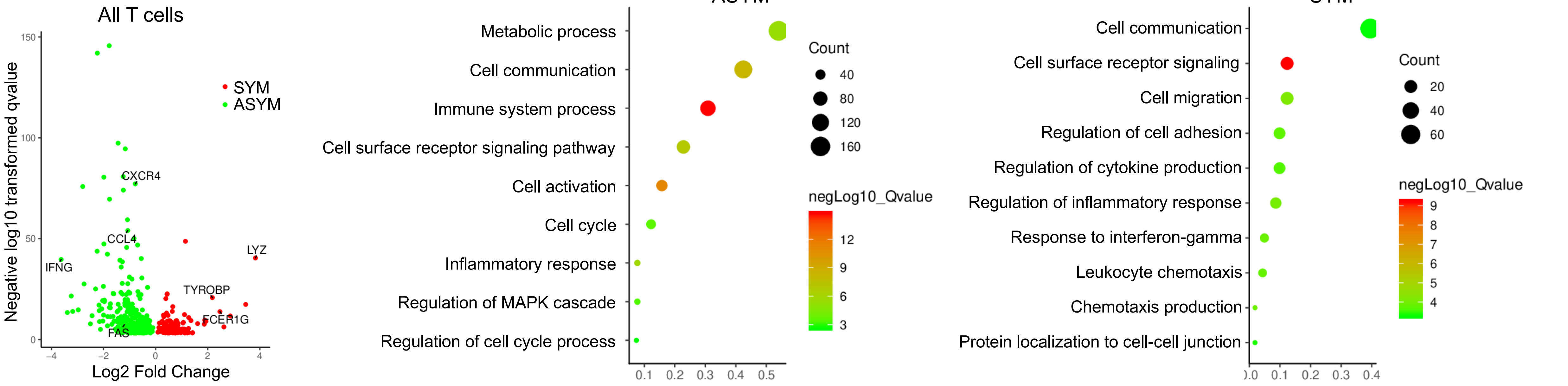
All T cells
Negative log10 transformed qvalue
Log2 Fold Change
SYM
ASYM
CXCR4
CCL4
IFNG
LYZ
TYROBP
FCER1G
FAS
150
100
50
0
−4
−2
0
2
4
ASYM
Metabolic process
Cell communication
Immune system process
Cell surface receptor signaling pathway
Cell activation
Cell cycle
Inflammatory response
Regulation of MAPK cascade
Regulation of cell cycle process
0.1
0.2
0.3
0.4
0.5
Count
40
80
120
160
negLog10_Qvalue
12
9
6
3
SYM
Cell communication
Cell surface receptor signaling
Cell migration
Regulation of cell adhesion
Regulation of cytokine production
Regulation of inflammatory response
Response to interferon-gamma
Leukocyte chemotaxis
Chemotaxis production
Protein localization to cell-cell junction
0.1
0.2
0.3
0.4
Count
20
40
60
negLog10_Qvalue
9
8
7
6
5
4

S4B

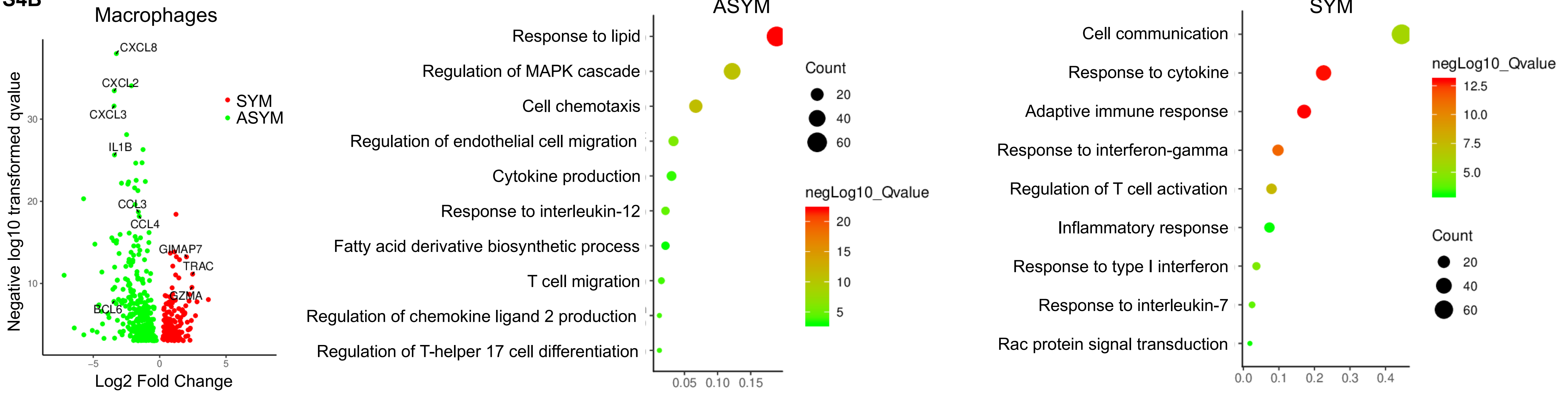
Macrophages
Negative log10 transformed qvalue
Log2 Fold Change
SYM
ASYM
CXCL8
CXCL2
CXCL3
IL1B
CCL3
CCL4
GIMAP7
TRAC
GZMA
BCL6
30
20
10
−5
0
5
ASYM
Response to lipid
Regulation of MAPK cascade
Cell chemotaxis
Regulation of endothelial cell migration
Cytokine production
Response to interleukin-12
Fatty acid derivative biosynthetic process
T cell migration
Regulation of chemokine ligand 2 production
Regulation of T-helper 17 cell differentiation
0.05
0.10
0.15
Count
20
40
60
negLog10_Qvalue
20
15
10
5
SYM
Cell communication
Response to cytokine
Adaptive immune response
Response to interferon-gamma
Regulation of T cell activation
Inflammatory response
Response to type I interferon
Response to interleukin-7
Rac protein signal transduction
0.0
0.1
0.2
0.3
0.4
negLog10_Qvalue
12.5
10.0
7.5
5.0
Count
20
40
60

S4C

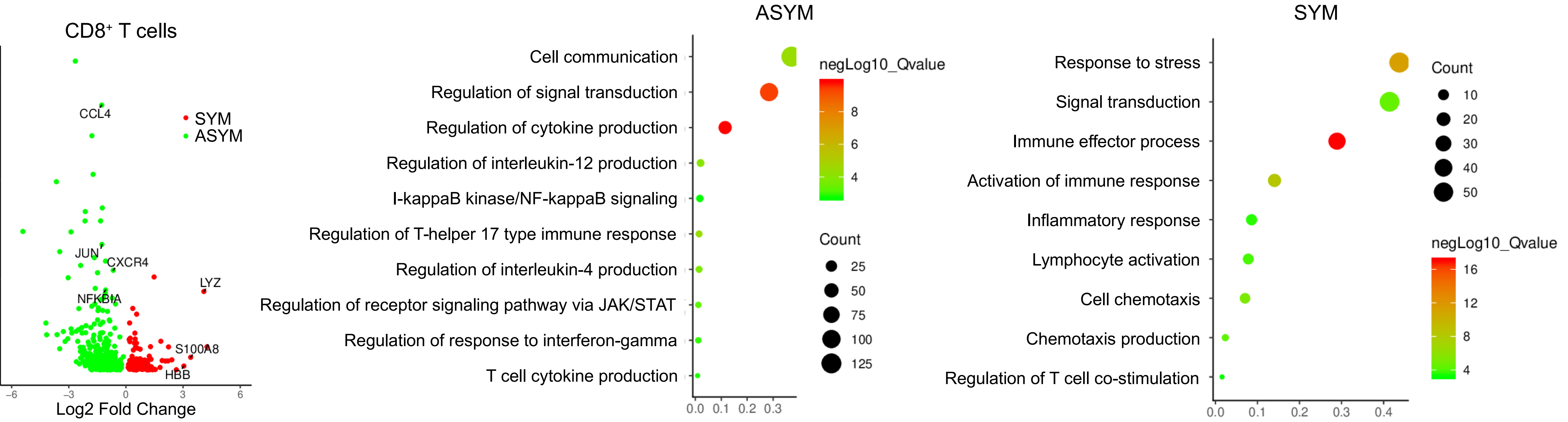
CD8⁺ T cells
Negative log10 transformed qvalue
Log2 Fold Change
SYM
ASYM
CCL4
JUN
CXCR4
LYZ
NFKBIA
S100A8
HBB
ASYM
Cell communication
Regulation of signal transduction
Regulation of cytokine production
Regulation of interleukin-12 production
I-kappaB kinase/NF-kappaB signaling
Regulation of T-helper 17 type immune response
Regulation of interleukin-4 production
Regulation of receptor signaling pathway via JAK/STAT
Regulation of response to interferon-gamma
T cell cytokine production
negLog10_Qvalue
Count
SYM
Response to stress
Signal transduction
Immune effector process
Activation of immune response
Inflammatory response
Lymphocyte activation
Cell chemotaxis
Chemotaxis production
Regulation of T cell co-stimulation
Count
negLog10_Qvalue

S4D

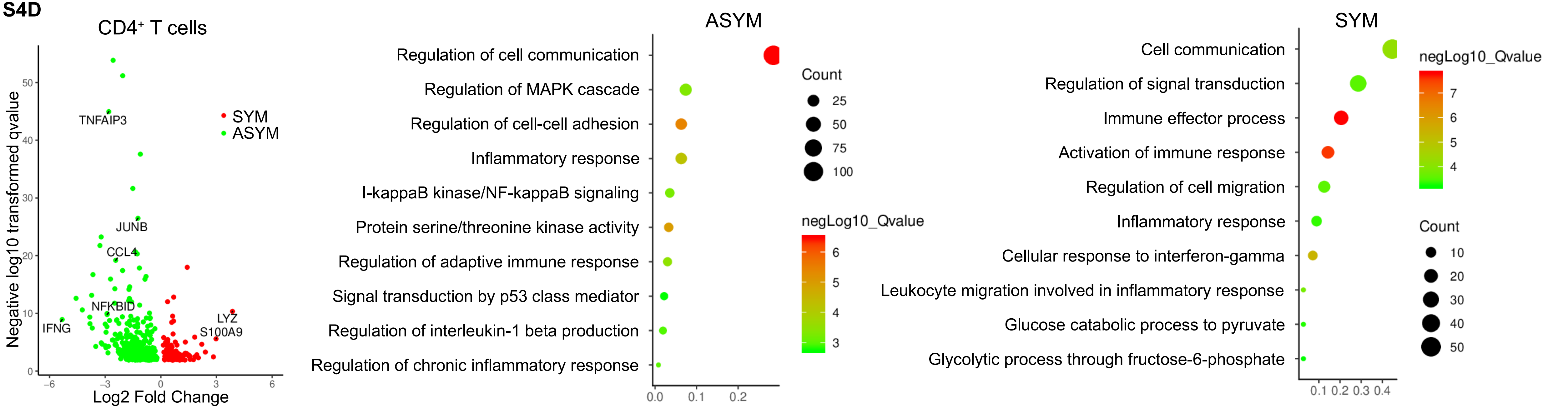
CD4⁺ T cells
Negative log10 transformed qvalue
Log2 Fold Change
SYM
ASYM
TNFAIP3
JUNB
CCL4
NFKBID
IFNG
LYZ
S100A9
ASYM
Regulation of cell communication
Regulation of MAPK cascade
Regulation of cell-cell adhesion
Inflammatory response
I-kappaB kinase/NF-kappaB signaling
Protein serine/threonine kinase activity
Regulation of adaptive immune response
Signal transduction by p53 class mediator
Regulation of interleukin-1 beta production
Regulation of chronic inflammatory response
Count
negLog10_Qvalue
SYM
Cell communication
Regulation of signal transduction
Immune effector process
Activation of immune response
Regulation of cell migration
Inflammatory response
Cellular response to interferon-gamma
Leukocyte migration involved in inflammatory response
Glucose catabolic process to pyruvate
Glycolytic process through fructose-6-phosphate
negLog10_Qvalue
Count

sFigure 4. gene expression of T cells in human with atherosclerosis

(A) Volcano plot showed selected differentially expressed genes in human and Gene Ontology pathway analysis. All T cells from ASYM and SYM.

(B) Volcano plot showed selected differentially expressed genes in human and Gene Ontology pathway analysis. macrophages from ASYM and SYM.

(C) Volcano plot showed selected differentially expressed genes in human and Gene Ontology pathway analysis. $CD8^+$ T cells from ASYM and SYM.

(D) Volcano plot showed selected differentially expressed genes in human and Gene Ontology pathway analysis. $CD4^+$ T cells from ASYM and SYM.

sFigure 5A

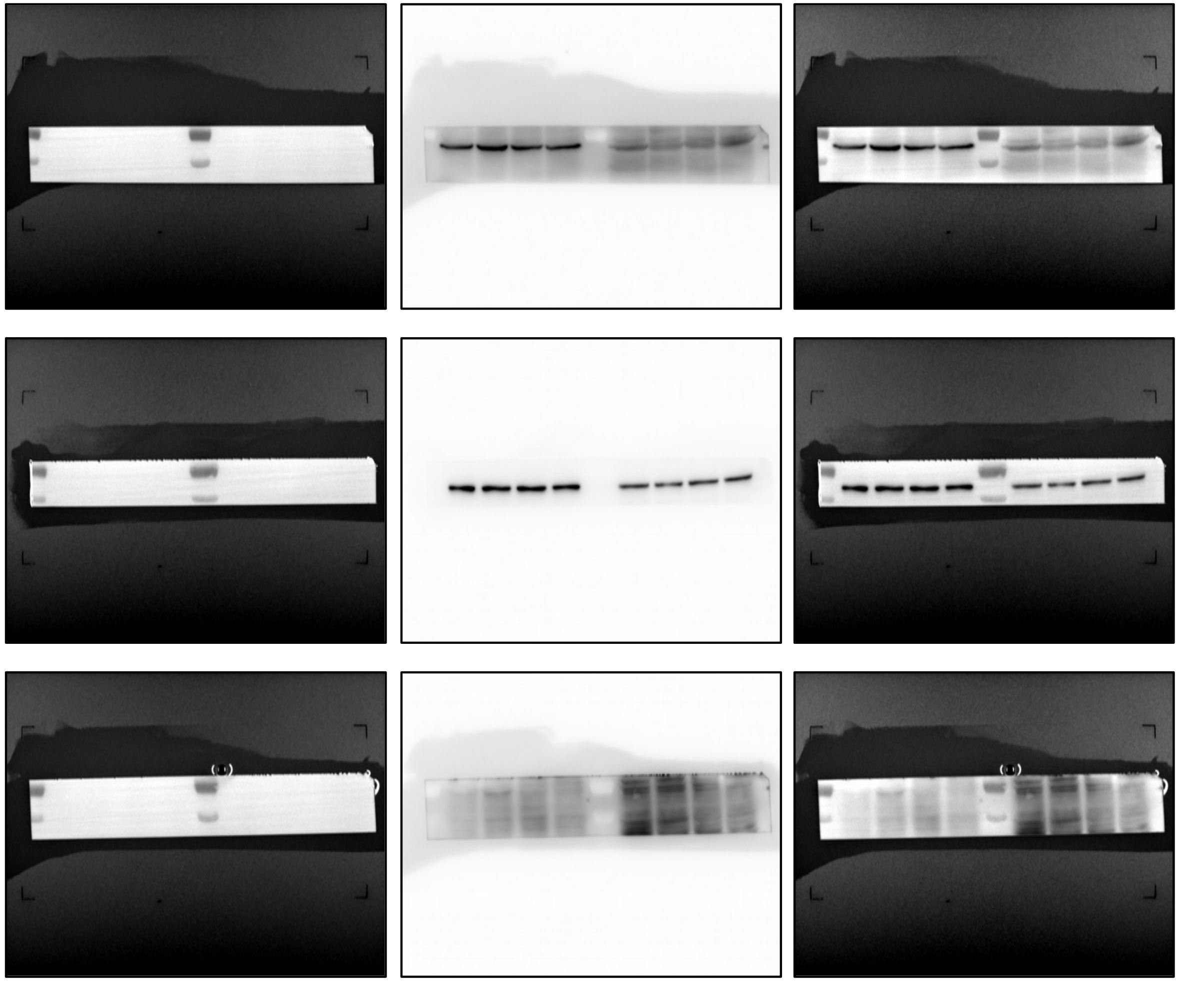

sFigure 5A. Uncropped images of the western blots presented in the Fig.5A
Maker: PageRuler™ Prestained Protein Ladder, 10 to 180 kDa (#26616, Thermo).
HRP: Images were acquired using Tanon 5200 by chemiluminescence function.
Merge: Combine the Marker and HRP images by Tanon software.

sFigure 5B

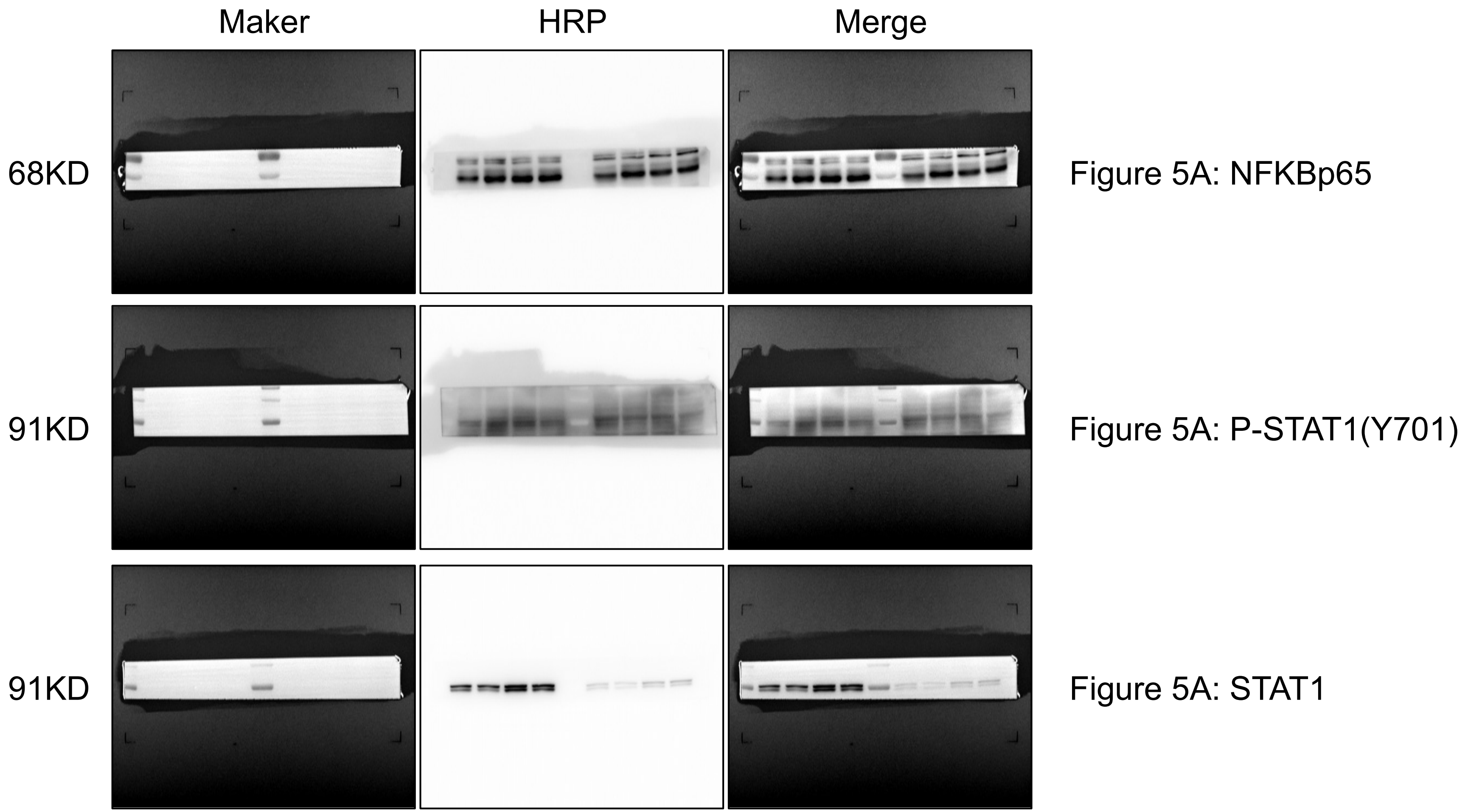

sFigure 5B. Uncropped images of the western blots presented in the Fig.5A
Maker: PageRuler™ Prestained Protein Ladder, 10 to 180 kDa (#26616, Thermo).
HRP: Images were acquired using Tanon 5200 by chemiluminescence function.
Merge: Combine the Marker and HRP images by Tanon software.

sFigure 5C

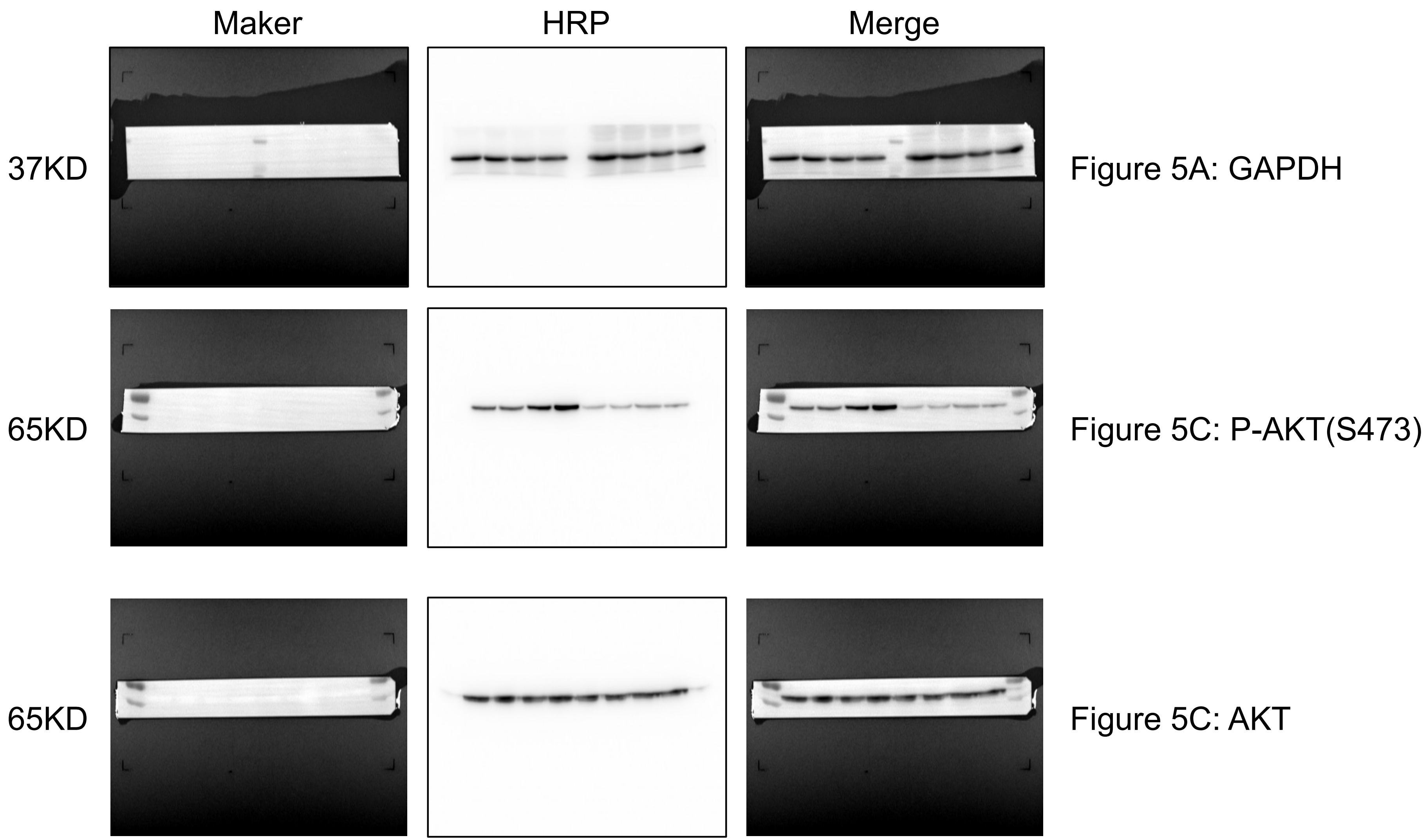

sFigure 5C. Uncropped images of the western blots presented in the Fig.5A & Fig. 5C
Maker: PageRuler™ Prestained Protein Ladder, 10 to 180 kDa (#26616, Thermo).
HRP: Images were acquired using Tanon 5200 by chemiluminescence function.
Merge: Combine the Marker and HRP images by Tanon software.

sFigure 5D

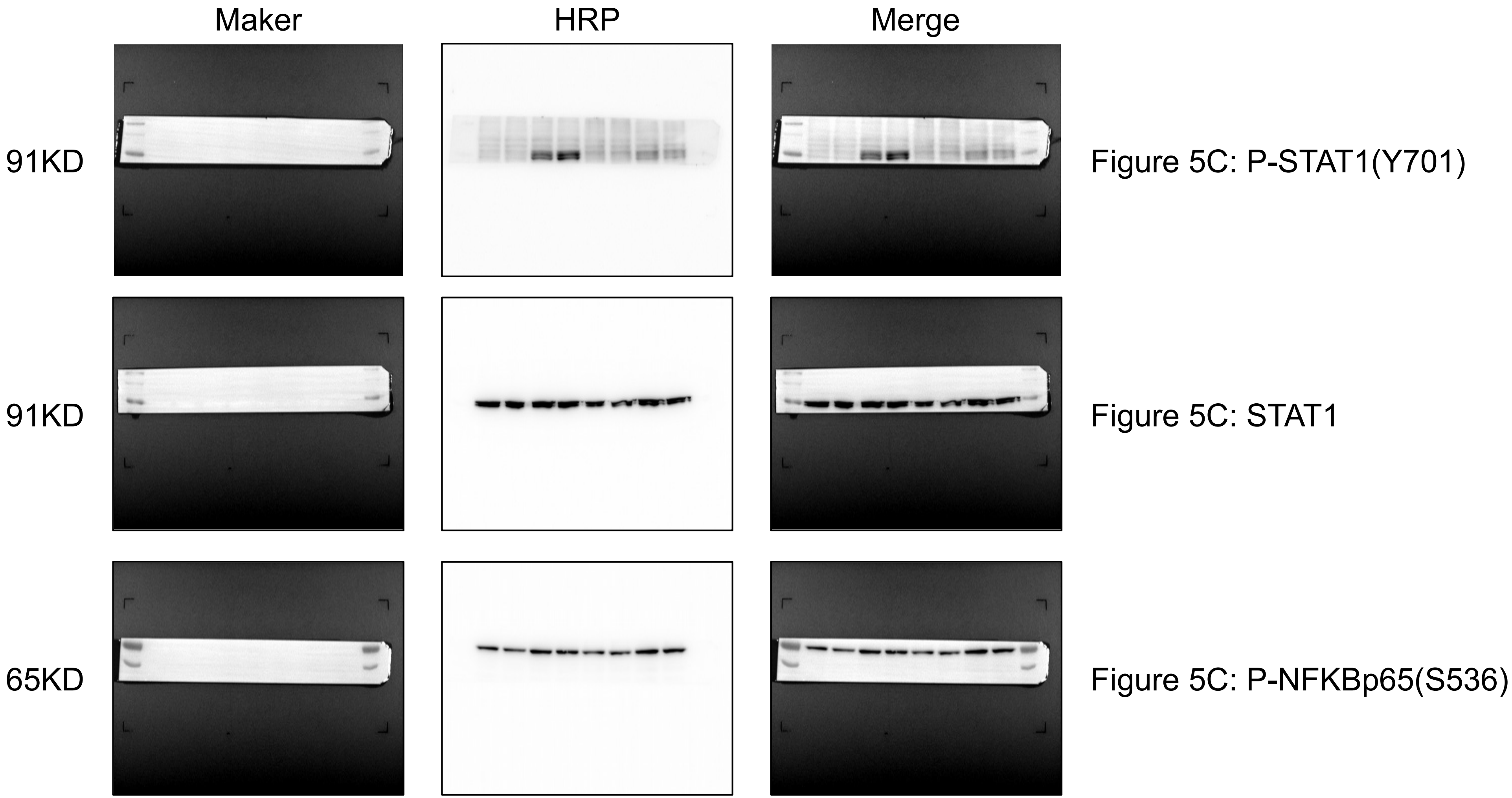

sFigure 5D. Uncropped images of the western blots presented in the Fig.5C
Maker: PageRuler™ Prestained Protein Ladder, 10 to 180 kDa (#26616, Thermo).
HRP: Images were acquired using Tanon 5200 by chemiluminescence function.
Merge: Combine the Marker and HRP images by Tanon software.

sFigure 5E

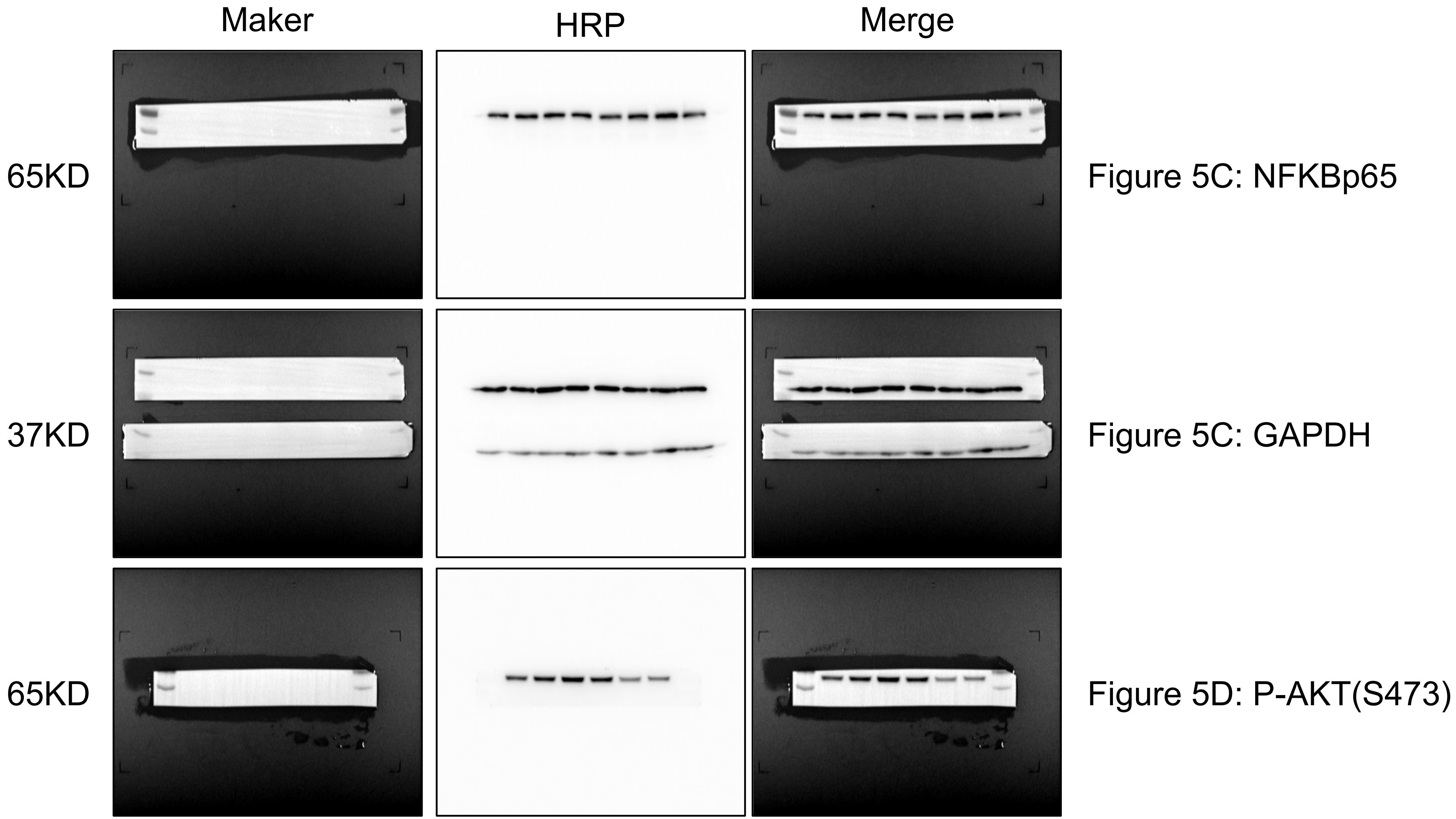

sFigure 5E. Uncropped images of the western blots presented in the Fig.5C & Fig.5D
Maker: PageRuler™ Prestained Protein Ladder, 10 to 180 kDa (#26616, Thermo).
HRP: Images were acquired using Tanon 5200 by chemiluminescence function.
Merge: Combine the Marker and HRP images by Tanon software.

sFigure 5F

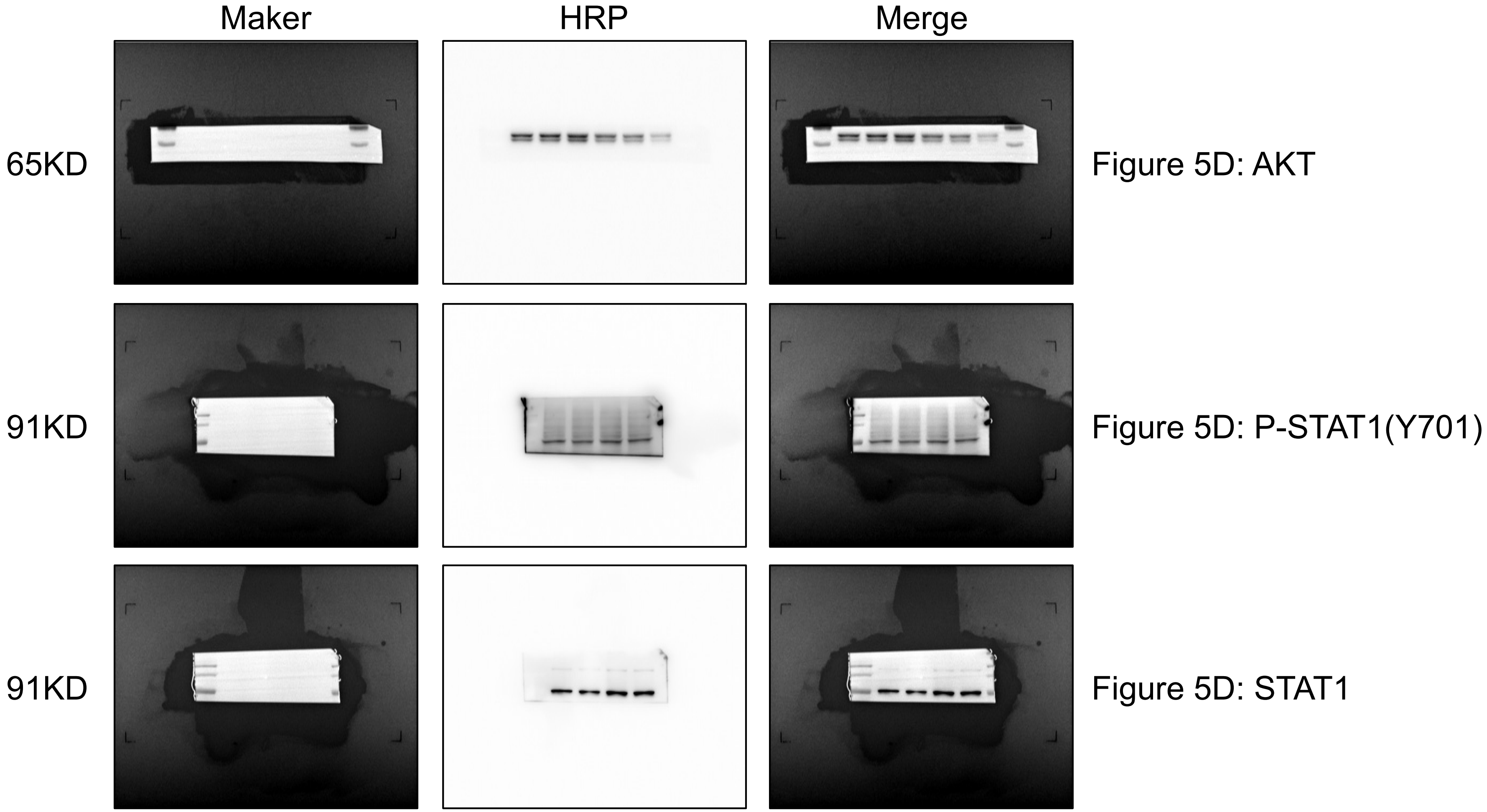

sFigure 5F. Uncropped images of the western blots presented in the Fig.5D
Maker: PageRuler™ Prestained Protein Ladder, 10 to 180 kDa (#26616, Thermo).
HRP: Images were acquired using Tanon 5200 by chemiluminescence function.
Merge: Combine the Marker and HRP images by Tanon software.

sFigure 5G

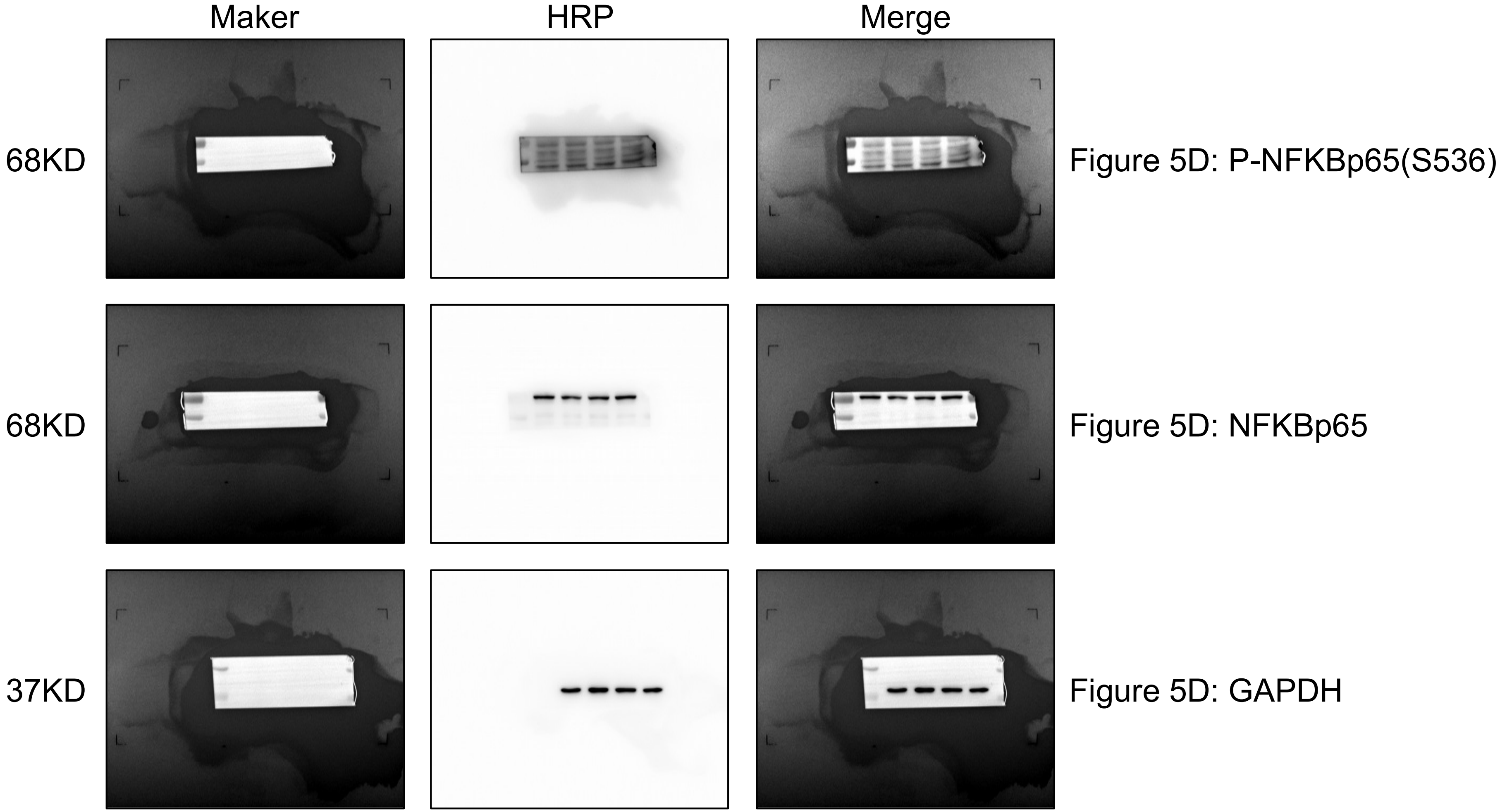

sFigure 5G. Uncropped images of the western blots presented in the Fig.5D
Maker: PageRuler™ Prestained Protein Ladder, 10 to 180 kDa (#26616, Thermo).
HRP: Images were acquired using Tanon 5200 by chemiluminescence function.
Merge: Combine the Marker and HRP images by Tanon software.

Table S1. Clinical characteristics of all patients from database.

Table S2. The proportion of cell types in the results of single cell sequencing.

Table S3. Top 500 Differentially Expressed Genes in T cells/CD8+/CD4+/Macrophages of patients (ASYM and SYM).

Table S4. Top 500 Differentially Expressed Genes in T cells/CD8+/CD4+ of SYM (plaques and blood)

Table S5. GO pathway analysis of T cells/CD8+/CD4+/Macrophages in patients (ASYM and SYM).

Table S6. GO pathway analysis of T cells/CD8+/CD4+ in SYM (plaques and blood).
